# CRISPR/Cas9-induced knockout and knock-in mutations in *Chlamydomonas reinhardtii*

**DOI:** 10.1038/srep27810

**Published:** 2016-06-13

**Authors:** Sung-Eun Shin, Jong-Min Lim, Hyun Gi Koh, Eun Kyung Kim, Nam Kyu Kang, Seungjib Jeon, Sohee Kwon, Won-Sub Shin, Bongsoo Lee, Kwon Hwangbo, Jungeun Kim, Sung Hyeok Ye, Jae-Young Yun, Hogyun Seo, Hee-Mock Oh, Kyung-Jin Kim, Jin-Soo Kim, Won-Joong Jeong, Yong Keun Chang, Byeong-ryool Jeong

**Affiliations:** 1 Department of Chemical and Biomolecular Engineering, Korea Advanced Institute of Science and Technology (KAIST), Daejeon 34141, Republic of Korea; 2 Korea Research Institute of Bioscience and Biotechnology (KRIBB), Daejeon 34141, Republic of Korea; 3 Advanced Biomass R&D Center (ABC), KAIST, Daejeon 34141, Republic of Korea; 4 Department of Biological Science, Chungnam National University (CNU), Daejeon 34134, Republic of Korea; 5 Center for Genome Engineering, Institute for Basic Science (IBS), Seoul 08826, Republic of Korea; 6 Department of Chemistry, Seoul National University (SNU), Seoul 08826, Republic of Korea; 7 Basic science, IBS school, Korea University of Science and Technology (UST), Seoul 08826, Republic of Korea; 8 School of Life Sciences, KNU Creative BioResearch Group, Kyungpook National University (KNU), Daegu 41566, Republic of Korea

## Abstract

Genome editing is crucial for genetic engineering of organisms for improved traits, particularly in microalgae due to the urgent necessity for the next generation biofuel production. The most advanced CRISPR/Cas9 system is simple, efficient and accurate in some organisms; however, it has proven extremely difficult in microalgae including the model alga *Chlamydomonas*. We solved this problem by delivering Cas9 ribonucleoproteins (RNPs) comprising the Cas9 protein and sgRNAs to avoid cytotoxicity and off-targeting associated with vector-driven expression of Cas9. We obtained CRISPR/Cas9-induced mutations at three loci including *MAA7*, *CpSRP43* and *ChlM*, and targeted mutagenic efficiency was improved up to 100 fold compared to the first report of transgenic Cas9-induced mutagenesis. Interestingly, we found that unrelated vectors used for the selection purpose were predominantly integrated at the Cas9 cut site, indicative of NHEJ-mediated knock-in events. As expected with Cas9 RNPs, no off-targeting was found in one of the mutagenic screens. In conclusion, we improved the knockout efficiency by using Cas9 RNPs, which opens great opportunities not only for biological research but also industrial applications in *Chlamydomonas* and other microalgae. Findings of the NHEJ-mediated knock-in events will allow applications of the CRISPR/Cas9 system in microalgae, including “safe harboring” techniques shown in other organisms.

Microalgae have emerged as the third generation biofuel feedstocks that are excellent renewable and sustainable energy sources for the future. Their use would allow us to replace the current fossil fuels that have been blamed for serious environmental problems, including pollution and global warming^1^. Microalgae have many advantages over the previous generations of biofuel feedstocks: they have much higher photosynthetic productivity, require less land, may not compete with food sources, and can generate more value-added products^2^. However, microalgal biofuels are currently impractical due to their high production costs. This can be partly solved by genetic improvements of microalgal strains to produce more biomass and/or lipids that can be converted to biofuels.

The green unicellular flagellate alga, *Chlamydomonas reinhardtii*, is a good model organism, with genetic, molecular and genomic resources available for both research fields of basic and applied sciences. Numerous techniques have been used to transform the nucleus, chloroplast and mitochondria of this microalga^3^,^4^,^5^, allowing genetic improvements of traits for various purposes. These advanced resources and techniques have been applied to other microalgae for more practical purposes: for example, transformation techniques have been used to improve production of biomass and/or biofuels in industrial microalgae by overexpression of certain metabolic or regulatory genes^6^,^7^. However, a remaining challenge in the genetic engineering of microalgae is the need for efficient methods to specifically knock-down or knockout unwanted genes.

Targeting specific genes for down-regulation or disruption has been well established in many organisms^8^,^9^,^10^,^11^,^12^. In microalgae, genes can be down-regulated by RNA silencing techniques involving RNA interference (RNAi) or artificial microRNAs (amiRNA)^13^,^14^,^15^,^16^,^17^. These techniques can be used to study essential genes, for which recessive mutations are lethal, particularly in haploid microalgae; however, these methods are often limited by low efficiency, non-specific targeting and silencing of the RNA constructs^9^,^18^. Stable and permanent disruption of unwanted genes can be achieved by homologous recombination (HR), and by sequence-specific recombinant nucleases including zinc-finger nuclease (ZFN) and transcription activator-like effector nuclease (TALEN)^11^,^12^. In some microalgae, only a few reports have described the specific disruption of genes via HR^19^,^20^ and ZFN- or TALEN-based knockout^21^,^22^,^23^; however, these techniques are very hard to achieve in most microalgae.

Recently, a new system based on components of the clustered regularly interspaced short palindromic repeats (CRISPR)/CRISPR associated protein 9 (Cas9), has become very popular in disrupting a gene due to its simplicity, improved specificity and higher versatility compared to ZFN and TALEN^24^,^25^. The CRISPR/Cas9 system was initially identified as a bacterial adaptive immune system against invading plasmids and viral DNAs, where the system was found to cleave target DNAs guided by small RNAs^24^,^26^. Since the introduction of CRISPR/Cas9 for heterologous genome editing^27^, a plethora of studies have used CRISPR/Cas9 to knockout targeted genes^28^. However, it has proven difficult to use this system for genome editing in microalgae. Only one such report exists for *Chlamydomonas*, and extremely low targeting efficiency was observed, possibly reflecting toxicity induced by vector-driven Cas9 expression^29^.

In this study, we improved the targeting efficiency of CRISPR/Cas9 in *Chlamydomonas* by directly delivering the Cas9 protein and single-chain guide RNAs (sgRNAs), known as Cas9 ribonucleoprotein (RNP), which has been successfully tested in other organisms^28^,^30^,^31^. We targeted three genes: the *MAA7* gene that encodes the beta subunit of tryptophan synthase (TSB); the antennal assembly gene *CpSRP43* that encodes chloroplast SRP43; and the chlorophyll biosynthetic gene *ChlM* that encodes Mg-protoporphyrin IX S-adenosyl methionine O-methyl transferase. Mutations of *MAA7* can be positively selected using 5-fluoroindole (5-FI)^32^,^33^, while mutations of *cpsrp43* and *chlm* can be selected by a lighter colony color due to reduced antenna size and chlorophyll synthesis, respectively^34^,^35^. For the latter two genes, we employed co-transformation of vectors that confer resistance to hygromycin due to low delivery efficiency in *Chlamydomonas*. Interestingly, the vectors used in our mutagenic screen were found to be integrated at the Cas9 cut site, indicating that the knock-in mutations were mediated by non-homologous end joining (NHEJ). In summary, we obtained many auxotrophic and visible colonies from the primary screen, and succeeded in obtaining sizable number of mutations targeted at the Cas9 cut sites.

## Results

### Design of sgRNAs and *in vitro* assessment of the Cas9 activity

To avoid the probable problems of toxicity^29^ and off-targeting^30^ that have been associated with vector-driven expression of the guide RNA and Cas9, we used synthetic sgRNAs and the Cas9 protein manufactured by ToolGen, Inc. (Republic of Korea)^30^ instead of cloning their genes in a vector. The sgRNAs for the individual target genes were designed to maximize the generation of mutations, as described in the Methods section as per the manufacturer’s guidelines (ToolGen, Inc., Republic of Korea). Target genes were chosen to facilitate easy selection of mutants. Mutants of the *MAA7* gene were identified via tryptophan auxotrophic selection using 5-FI^32^,^33^, whereas mutations in *CpSRP43* and *ChlM* were identified by visual selection due to the reduced chlorophyll contents of these mutants^34^,^35^.

**Figure 1:**
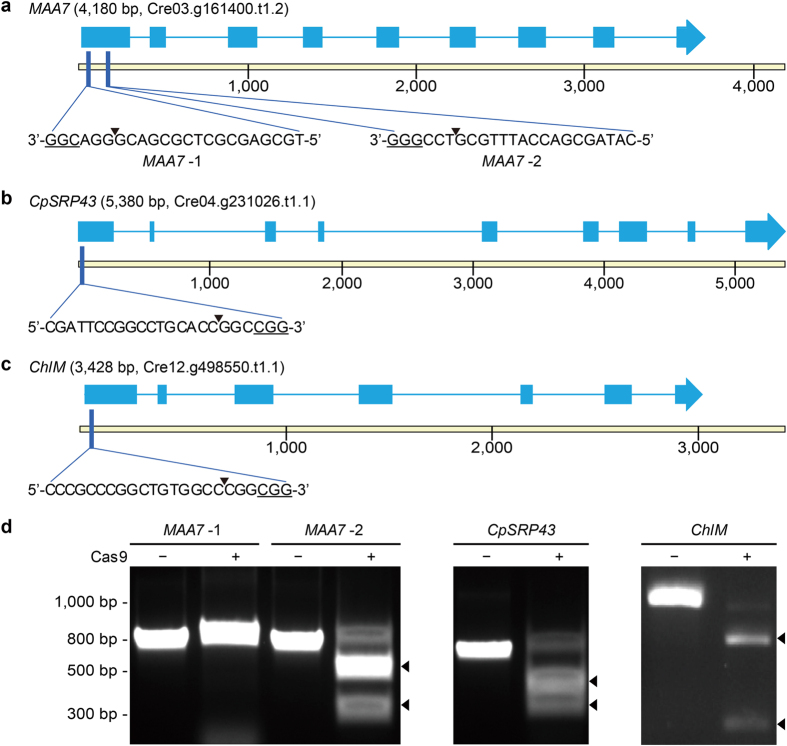
Map of CRISPR/Cas9 target sites in the *MAA7*, *CpSRP43* and *ChlM* loci, and *in vitro* cleavage of target DNAs by sgRNAs and the Cas9 protein. (**a**–**c**) Molecular maps of the *MAA7* (**a**), *CpSRP43* (**b**) and *ChlM* (**c**) loci with the CRISPR/Cas9 target sites indicated. CDS sequences are indicated as blue boxes, together with the sgRNA target sequences, the cut sites (arrowheads) and the associated PAM sites (5′-NGG-3′, underlined). Target sites of *MAA7* were written in the 3′-to-5′ orientation, since the negative strand was targeted by sgRNAs. (**d**) The activities of the Cas9 protein and individual sgRNAs were tested *in vitro*. The Cas9-degraded products of the amplified fragments are indicated by arrowheads, with degraded products of 488 and 295 bp, 418 and 377 bp, and 736 and 274 bp identified for *MAA7*, *CpSRP43* and *ChlM*, respectively.

The activities of the sgRNAs and Cas9 protein were assessed by *in vitro* reactions using individual genomic fragments containing the Cas9 cut site (Fig. 1). The primers used to amplify the relevant genomic fragments are summarized in Table S1. Figure 1 summarizes the genomic maps, the locations and sequences of the guide RNAs, the protospace adjacent motifs (PAMs) and the Cas9 cut site for *MAA7*, *CpSRP43* and *ChlM*. We tested two sgRNAs for *MAA7* and found that the first (*MAA7*-1) failed to cleave, while the second (*MAA7*-2) succeeded in cleaving the genomic DNA target of 783 bp into two fragments of 488 bp and 295 bp (Fig. 1d left panel). The *CpSRP43* Cas9 RNP cleaved the 795 bp target DNA into 418 bp and 377 bp fragments, while the *ChlM* Cas9 RNP cleaved the targets of 1010 bp into 274 bp and 736 bp fragments (Fig. 1d right panels). Consistent with these *in vitro* results, our later *in vivo* mutagenic screens failed to isolate any targeted mutations for the *MAA7*-1 sgRNA (See below, and Table 1).

### Small indel mutations found among the *MAA7* auxotrophic mutants

**Figure 2:**
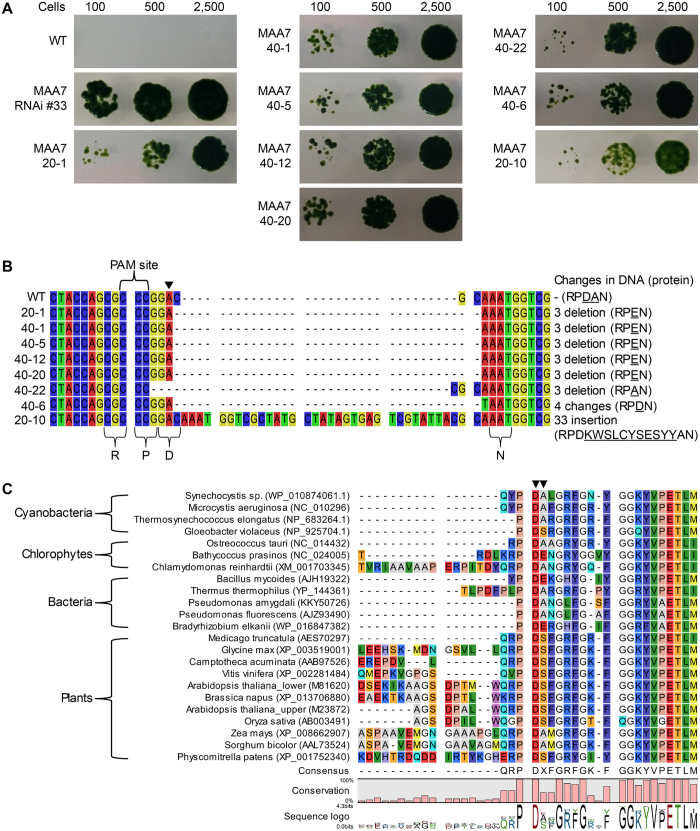
Auxotrophic selection of *MAA7* mutants and their sequence analyses. (**A**) The isolated *maa7* mutants were grown in the presence of 5-FI with tryptophan supplementation, and cell growth was examined by spot-test loading of 100, 500, and 2500 cells. The RNAi mutant, *MAA7* RNAi #33, was used as a reference strain. (**B**) Small indels are produced by CRISPR/Cas9 at the Cas9 cut site of the *MAA7* locus. Sequencing of genomic DNA fragments encompassing the cut site revealed eight small indels at the cut site (at the 3′ side of the arrow head). The protein sequences of the target region (the RPDAN motif) are listed on the right side, and the altered sequences are underlined. (**C**) Alignments of the N-terminal side of the TSB sequences from various organisms representing three kingdoms. GenBank IDs are included to facilitate identification. Mutated sequences are marked with arrowheads.

*In vivo* mutagenesis was carried out by electroporating *MAA7* Cas9 RNPs into *C. reinhardtii* CC-124. To optimize the delivery and mutagenic efficiencies, we performed experiments in which the amounts of sgRNA and Cas9 were varied while the mass ratio was kept constant at 4:3, as suggested by ToolGen, Inc. (Table 1). Selection with 5-FI yielded three and 21 tryptophan auxotrophic mutants from experiments performed using the *MAA7*-1 and *MAA7*-2 sgRNAs, respectively. The growth of some of these primary auxotrophic mutants on media containing tryptophan and 5-FI is shown in Fig. 2A. A *MAA7* RNAi mutant (*MAA7* RNAi #33) was used as a reference strain^17^,^36^. Our mutant strains all showed comparable tolerances to 5-FI, but were consistently less tolerant than *MAA7* RNAi #33 (Fig. 2A). Genomic DNA was amplified using primers M2_F and M2-R (Table S1), and sequencing of the generated fragments revealed that zero and eight targeted mutations had been obtained using the *MAA7*-1 and *MAA7*-2 sgRNAs, respectively (Table 1, Fig. 2B). Therefore, as noted above, the *MAA7*-1 sgRNA failed to cleave the target DNA *in vitro* and produced no mutations *in vivo*. It is not clear why we obtained such different outcomes from the two target sites closely located on the same locus. However, our results suggest that it is important to test the compatibility of the sgRNA and Cas9 protein prior to undertaking a laborious mutagenic screen.

Sequencing of the eight targeted mutations revealed an interesting pattern of small indel mutations that involved three base pairs or multiples. This yielded point mutations of only a few amino acids rather than the frameshift mutations commonly found in CRISPR/Cas9-induced mutations in other organisms. Our targeted mutations could be grouped into four types. The main type (found in mutants of *MAA7* 20-1, 40-1, 40-5, 40-12 and 40-20; mutant designation indicated amount sgRNA followed by a serial number.) was deletion of the CGC next to the Cas9 cut site producing a D54E point mutation and a deletion of A55. For convenience, we defined a conserved motif, RPDAN, flanking the Cas9 cut site (Fig. 2B); it was changed to RPEN in these mutants. The other three mutation types were alteration of the RPDAN motif to RPAN (*MAA7* 40-22), alteration of the RPDAN motif to RPDN (*MAA7* 40-6), and a 33 bp insertion at the Cas9 cut site (*MAA7* 20-10) that changed the RPDAN motif to RPDKWSLCYSESYYAN via insertion of the 11 underlined amino acids.

To assess the significance of the RPDAN motif and validate our CRISPR/Cas9-induced mutagenic screen using 5-FI, we performed additional experiments including phylogenetic analyses of TSB homologs, sequencing of *MAA7*-associated genes, and complementation of *MAA7* mutants. Phylogenetic analyses of TSB homologs from various organisms representing the kingdoms of bacteria, plants and microalgae revealed that the RPDAN motif was a part of a conserved region found in all organisms (Fig. 2C; Fig. S1 for the full alignment; Fig. S2 for the phylogenetic tree). The R52 residue was conserved in plants and microalgae, P53 and D54 were invariable in all studied organisms, and A55 appeared to be conserved in bacteria and microalgae. Bacteria and microalgae that shared conservation of A55 appeared to share aspects of the entire sequence phylogeny, as they grouped together in the phylogenetic tree (Fig. S2). The RPDAN motif is clearly distant from the catalytic site of TSB^33^. We suggest that it may be involved in the interaction with tryptophan synthase alpha subunit (TSA), based on our structural modeling of TSA and TSB in bacteria (Fig. S3). When we compared the reported bacterial TSA-TSB structures with the amino acid sequence of *MAA7*, we found that the RPDAN motif was located proximal to the four-stranded β-sheet that is involved in the interaction between TSA and TSB (Fig. S3). Because the interface between the two subunits is crucial for the necessary consecutive enzyme reactions^37^, we speculate that the mutation-based impairment of interface might reduce the enzyme activity of the protein. To exclude the possibility that this second locus might be involved in the suppression during the auxotrophic selection, we sequenced the genes encoding TSA and TSB in WT and mutants *MAA7* 20-1, 40-22, 40-6 and 20-10 (representing all four mutation classes). We did not identify any mutations other than those at the Cas9 cut site as shown in Fig. 2B. Alternatively, the RPDAN motif might be involved in other critical functions such as stability of the protein, which may explain the low expression of mutated TSB (Fig. S4c).

### Complementation of the *MAA7* mutations

We further analyzed the functional significance of the RPDAN motif *in vivo* by performing complementation analyses. The TSB coding sequences (CDSs) were amplified from WT and the *MAA7* 20-1 mutant (containing RPEN instead of the RPDAN motif), cloned into the pCr202 vector^38^ together with sequences encoding the FLAG tag, and transformed into *MAA7* RNAi #33 (Fig. S4a). The WT TSB CDS was also transformed into *MAA7* 20-1 to see if the defective TSB function of this mutant could be complemented by WT TSB. PCR was used to confirm transformation (Fig. S4b), and the expression of the TSB proteins tagged with the FLAG tag was assessed by Western blotting using an anti-FLAG antibody (Fig. S4c). All strains complemented with WT TSB (designated 20 W for the *MAA7* 20-1 mutant complemented with the WT TSB; RW for *MAA7* RNAi #33 transformed with the WT TSB) showed relatively high level of TSB expression. For unknown reasons, strains complemented with the mutagenized TSB CDS from *MAA7* 20-1 showed relatively lower expression levels.

**Figure 3:**
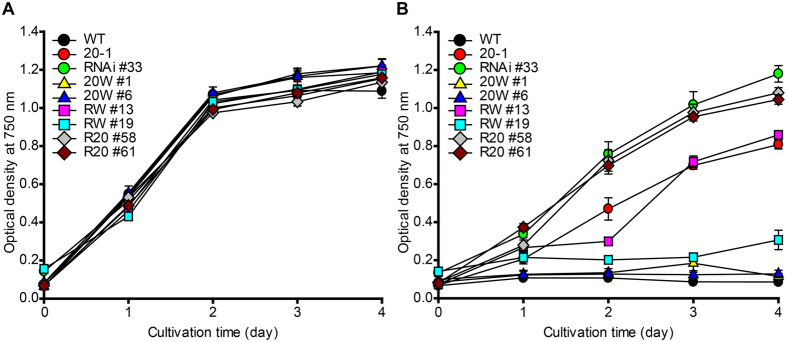
Complementation of *MAA7* mutants with WT and *MAA7* 20-1 TSB sequences. The *MAA7* 20-1 mutant was transformed with WT *MAA7* gene for complementation (generating strain 20W). RNAi #33 (a reference strain with a RNAi knockdown mutation of *MAA7*) was transformed with the WT (generating RW) or 20-1 (generating R20) *MAA7* genes. (**A**) Cell growth was analyzed in TAP medium supplemented with tryptophan. (**B**) The tolerance of each strain to 5-FI was analyzed by culturing cells in TAP medium containing both 5-FI and tryptophan. Error bars indicate standard deviations obtained from four independent experiments.

Complementation was evaluated by analyzing the sensitivity to 5-FI (Fig. 3). All strains used in the complementation assay showed normal growth when cultured in TAP medium supplemented with tryptophan (Fig. 3A). In the presence of 5-FI, *MAA7* RNAi #33 transformed with WT TSB showed almost complete complementation when WT TSB expression was high (RW #19), but incomplete complementation when low WT TSB expression was low (RW #13). This dose dependence appears to be reasonable considering that higher level of WT TSB expression can counteract the RNA silencing in *MAA7* RNAi #33. WT TSB also completely complemented *MAA7* 20-1 as shown in 20 W #1 and 20 W #6 (Fig. 3B). This indicates that the defects found in *MAA7* 20-1 (Fig. 2A) arose solely from the RPEN mutation of the RPDAN motif, since no other mutation was found in the genes encoding TSA or TSB.

The results obtained from complementation with the TSB CDS containing point mutations in the RPDAN motif provide additional evidence for the functional importance of this motif. We transformed *MAA7* RNAi #33 with the TSB CDS from *MAA7* 20-1, which contained the RPEN mutation of the RPDAN motif (designated R20 #58 and R20 #61). Compared to the strains complemented with WT TSB (RW #13 and RW #19), these R20 strains completely failed to complement the RNAi mutation (Fig. 3B), even though the expression of TSB-FLAG in these strains was low (Fig. S4c). Nevertheless, this result suggests that the RPEN mutant protein lacks a significant portion of the WT TSB function. Notably, mutations in the RPDAN motif were weaker than the RNAi knockdown mutation (*MAA7* RNAi #33), as their tolerance to 5-FI was lower than that of *MAA7* RNAi #33 (Figs 2A and 3B).

### NHEJ-mediated knock-in mutations at the CpSRP43 locus

We tested our CRISPR/Cas9 systems for two more genes that affect the chlorophyll contents allowing visual selection of their mutants. Considering the extremely low delivery efficiency of transgenic materials in *Chlamydomonas*, we co-transformed vectors carrying a selection maker. We were thus able to first select transformed colonies, and then identify mutants by visual selection of lighter green colonies. We targeted *CpSRP43* by delivering *CpSRP43*-specific sgRNA (Fig. 1b,d), Cas9 protein and *Xba*I-linearized pCr202 vector. Our initial screen yielded 283 colonies that showed resistance to hygromycin; from them, we selected three colonies that stably showed a lighter green coloration. *CpSRP43* is involved in antennal protein assembly and its mutations are well known. We obtained two deletion mutants, CC-4561 and CC-4562^34^, from the Chlamydomonas Resource Center, and used these as reference strains. We also included two negative controls: CpSRP43 20-1 was from the same screen but showed a normal green colony color; vec-2 was from a vector-only transformation with no sgRNAs or Cas9, and had a normal green colony color.

**Figure 4:**
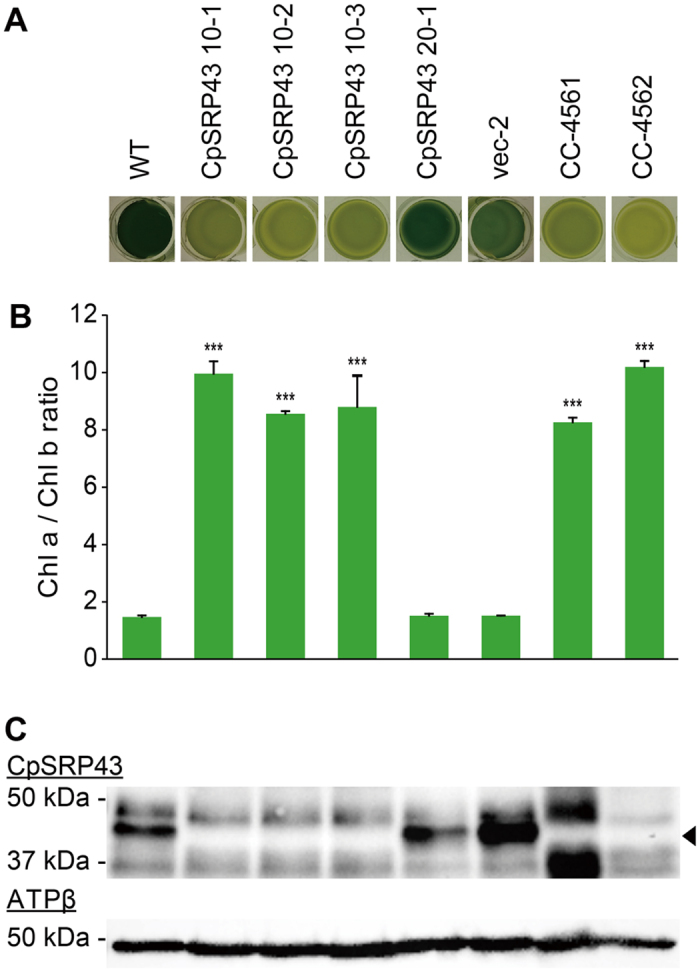
Phenotypic characterization of CpSRP43 mutants. Cells were co-transformed with sgRNA, the Cas9 protein and the selection vector, and three colonies showing a stable lighter green color were isolated and designated CpSRP43 10-1, 10-2 and 10-3. The negative controls included a colony from the same screen that carried the normal green color (designated CpSRP43 20-1), and a colony from an experiment in which cells were transformed with only the selection vector (designated vec-2). The positive controls included CC-4561 (mt+) and CC-4562 (mt−), which are deletion mutants of *CpSRP43* (also called *tla3-cpsrp43*). (**A**) The coloration of control colonies and those of CpSRP43 mutants induced by CRISPR/Cas9. Cells (2.50 × 10^7^ cells/mL) were loaded to a 24-well plate in identical volumes of TAP medium. (**B**) Changes in the chlorophyll contents of CpSRP43 mutants. The contents of chlorophyll a and b were measured at 72 hours of cultivation, normalized by the cell density. The ratios of chlorophyll a to chlorophyll b were calculated and plotted. Error bars indicate standard deviations obtained from four independent experiments. Significant differences compared to the WT were determined by the Student’s *t* test and are indicated by asterisk (*P < 0.05, **P < 0.01, ***P < 0.001) (**C**) Immunoblots were probed with antibodies against the target protein (CpSRP43) and the β-subunit of ATP synthase (ATPβ). A band of the molecular mass expected for the former (43 kDa) is marked with an arrowhead.

Our phenotypic analyses began with a visual assessment in which we loaded the same number of cells to each well of a multi-well plate, and compared their colors (Fig. 4A). Consistent with the colors observed for their colonies, the isolated candidates of *cpsrp43* mutants induced by CRISPR/Cas9 (CpSRP43 10-1, 10-2 and 10-3) were light green, similar to the *cpsrp43* deletion mutants CC-4561 and CC-4562. In contrast, the WT control, CC-124, and the other negative controls were dark green (Fig. 4A). We further analyzed the chlorophyll contents of these strains, and calculated the ratio of chlorophyll a to chlorophyll b, which is a simple criteria used for reduced antenna mutants^39^,^40^. As expected from their lighter green color, the chlorophyll contents of the candidate mutants were low, and their ratios of chlorophyll a to chlorophyll b were higher than 8, similar to the deletion mutants (Fig. 4B). Critically, when Western blotting was performed with an anti-CpSRP43 antibody, our CRISPR/Cas9-induced mutants and the deletion mutants all lacked the CpSRP43 protein with the expected size of 43 kDa (Fig. 4C). These data indicate that CpSRP43 sgRNA and Cas9 successfully induced mutations at the *CpSRP43* locus.

**Figure 5:**
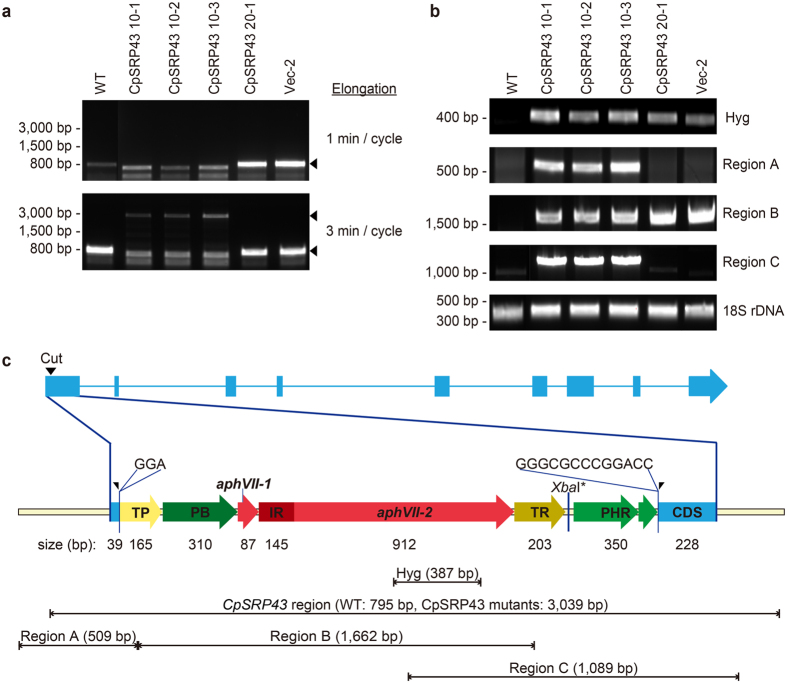
NHEJ-mediated knock-in of the co-transformed vector at the CRISPR/Cas9 cut site in the CpSRP43 locus. (**a**) PCR amplification of the *CpSRP43* regions was performed using different elongation times. Amplification of fragments encompassing the cut site failed under a short elongation cycle (upper panel), but improved when we used a longer cycle (lower panel). (**b**) Various vector sequences were confirmed by PCR. The amplicon sizes of the fragments encompassing the hygromycin resistance region, region A, region B, region C, and the 18S rDNA were 387 bp, 509 bp, 1,662 bp, 1,089 bp and 380 bp, respectively. The specific primers used in this study are listed in Table S1. (**c**) Molecular map of the *CpSRP43* locus and the integrated vector sequences. An insertion of 3 bp (GGA) was identified next to the 5′ side of the Cas9 cut site (left-half arrowhead). Considerable vector sequences (2,650 bp) were inserted at the cut site, followed by an additional 13 bp of unknown origin (GGGCGCCCGGACC) at the 3′ side of the cleaved site (right-half arrowhead). All of the *CpSRP43* mutants (10-1, 10-2 and 10-3) contained the same sequences at the Cas9 cut site in the *CpSRP43* locus. Abbreviations: TP, PsaD terminator; PB, beta2-tub promoter; IR, RBCS2 intron; TR, RBCS2 terminator; and PHR, HSP70A-RBCS2 promoter.

The molecular nature of the CRISPR/Cas9-induced mutants was analyzed with PCR using primers SRP43_2F and SRP43_2R (Table S1) with the expected size of 795 bp encompassing the Cas9 cut site. Initially, we failed to detect any amplification of the relatively small fragments with exception of a few fragments that were found to be nonspecific upon sequencing (Fig. 5A top panel). However, all of the transformants contained the vector sequence for the hygromycin resistance gene (Fig. 5B). Repeated PCR experiments using primer pairs spanning different areas of the locus yielded similar results. We thus sequenced their genomes, and found that the vector sequence was integrated at the Cas9 cut site with small insertions of a few nucleotides at either end (Fig. 5C). The insertion was further verified with PCR using different primer pairs (amplifying regions A, B and C) (Fig. 5B). Using optimized PCR conditions, we successfully amplified the whole *CpSRP43* locus in the mutants (Fig. 5A bottom panel). Assembly of all sequence information revealed that the vector sequence had been re-organized prior to integration as shown in Fig. 5C. All three mutants contained the same integrated sequence, suggesting that they arose from a single transformation event. We also characterized their integration in the genome by performing the Southern blot, and found that the integration was a single copy insertion with the same size of restriction fragments (Fig. S5A,B).

Off-targeting of the CpSRP43 Cas9 RNP was assessed in our mutagenic screen by using Cas-OFFinder (http://www.rgenome.net/cas-offinder/)^41^. The guide sequence was searched on the *Chlamydomonas* reference genome sequence allowing 3 mismatches and 2 bulges, and a total of 404 off-target candidate sites could be identified. Among these, we were able to locate 340 sites on our WT (CC-124) genomic sequence that matched those of the *Chlamydomonas* reference sequence (CC-503)^4^. We surveyed these 340 sequences on the genomic sequences of CpSRP43 10-1, 10-2 and 10-3, and confirmed the presence of 333 sites. These sites contained no mutations, suggesting that there were no, or rare, incidences of off-targeting with the CpSRP43 Cas9 RNP. This may be possible because the Cas9 RNP could have been degraded and/or diluted soon after electroporation.

### NHEJ-mediated knock-in mutations at the *ChlM* locus

**Figure 6:**
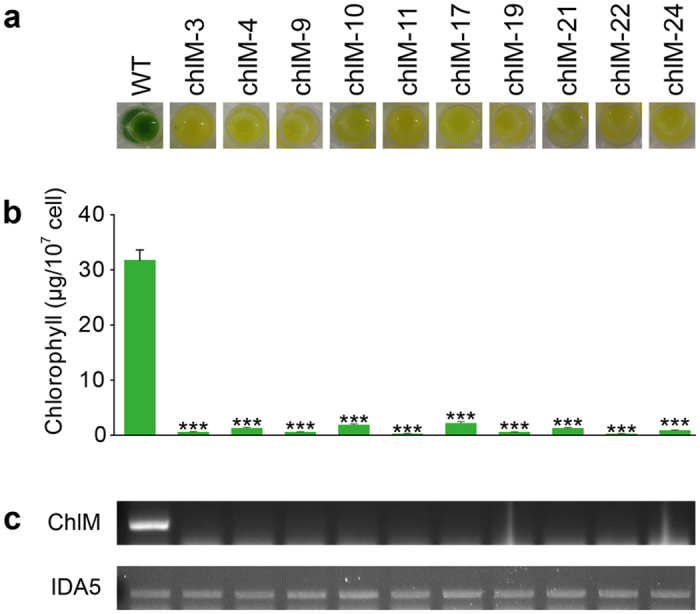
Phenotypic characterization of light green mutants induced by CRISPR/Cas9 at the *ChlM* locus. (**a**) Coloration of 10 ChlM mutants (2.0 × 10^8^ cells/mL) loaded to a 24-well plate in liquid TAP medium. (**b**) Chlorophyll contents of the ChlM mutants. Data are expressed as ± SD (n = 4 replicates). Significant differences compared to the WT control were determined by the Student’s *t* test and are indicated by asterisks (*P < 0.05, **P < 0.01, ***P < 0.001). (**c**) Semi-quantitative RT-PCR analysis was used to detect ChlM transcripts from the mutant lines. IDA5 was detected as a loading control.

Finally, we used our optimized CRISPR/Cas9 delivery conditions to mutate another gene, *ChlM*, involved in chlorophyll biosynthesis^35^. Vector pCr112 containing the hygromycin resistance marker was co-transformed for selection purpose. We obtained about 6000 initial transformants. From them, we isolated 10 clones that showed yellow or brown colony color, and designated ChlM-3, -4, -9, -10, -11, -17, -19, -21, -22, -24 (Fig. 6). Visual assessment of coloration (Fig. 6a) and measurement of chlorophyll contents (Fig. 6b) revealed that the mutant phenotype was associated with the expected chlorophyll deficiency^35^. We performed semi-quantitative RT-PCR, and found no detectable expression of ChlM mRNA in the mutants (Fig. 6c). Southern blot analyses were performed to characterize genomic integration patterns after digestion of their genomic DNAs with *Nco*I that does not cut the vector (Fig. S5c,d). Most of the selected transformants contained a single copy of the vector except for ChlM-4 that contained two copies. Two pairs of clones (ChlM-10 and ChlM-17; ChlM-22 and ChlM-24) showed the same banding patterns, suggesting that they were duplicate clones representing the same integration events. We thus observed at least eight independent integration events, most of which were single-copy integration of the vector.

**Figure 7:**
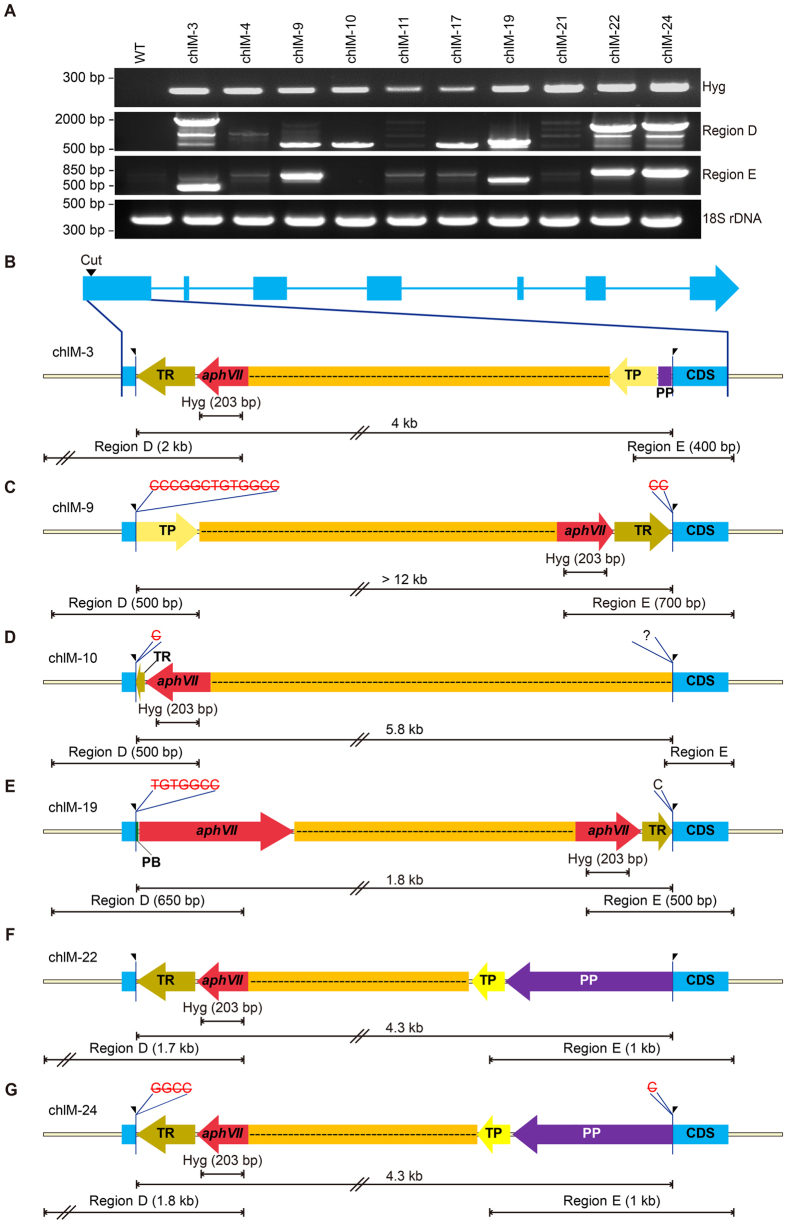
NHEJ-mediated knock-in mutants of the co-transformed vector at the CRISPR/Cas9 cut site on the *ChlM* locus. (**A**) Multiple PCR fragments encompassing the vector sequences. (**B**–**G**) Schematic molecular maps of the knock-in events in each ChlM mutant. The sizes of the inserted vector and PCR amplification are shown. Red and black letters indicated the deleted and inserted sequences, respectively. Abbreviations: TR, RBCS2 terminator; TP, PsaD terminator; PP, PsaD promoter; and PB, beta2-tub promoter.

We further characterized the molecular nature of the CRISPR/Cas9-induced mutations at the *ChlM* locus (Fig. 7). The initial PCR for the *ChlM* locus spanning the Cas9 cut site failed to amplify genomic DNA from the isolated mutants. We tested combinations of primers (Table S1) located in the *ChlM* locus and the vector, and successfully determined the junction sequences for six clones including ChlM-3, -9, -10, -19, -22 and -24 (Fig. 7B–G). All of them showed integration of the vector at the Cas9 cut site, and were characterized by different rearrangements of vector sequences and different small indels next to the Cas9 cut site. ChlM-22 and ChlM-24 showed the same vector sequence, but contained different deletions at the junctions (Fig. 7F,G). Our results indicate that the 10 isolated mutants represent at least nine independent integration events with extensive rearrangements of the vector sequences. Interestingly, the six clones with identified junction sequences were examples of NHEJ-mediated knock-in of the vector, because the vector contained no sequences homologous to the *ChlM* locus. We failed to identify the integration junction sequences for the remaining four clones; however, ChlM-4, ChlM-11 and ChlM-17 showed the same bands hybridized to both probes of *ChlM* and *aphVII*, suggesting that they are also knock-ins of the vector sequence. Only ChlM-21 showed different bands for the *ChlM* and *aphVII* probes, suggesting that the integration occurred at a location outside of the *ChlM* locus. ChlM-21 showed a smaller band for the *ChlM* probe compared to that of WT, suggesting that it contained a sizable deletion of about 200 bps (Fig. S5c,d). The nature of ChlM-21 has not been analyzed further, even though we speculated that there was a deletion of about 200 bps in the ChlM locus based on the Southern blot (Fig. S5c,d). It should be noted that ChlM-4 contained two copies of vector sequences: one at the *ChlM* locus and the other at an unknown location, based on the Southern blot (Fig. S5d). These mutations could be categorized into three classes as summarized in Fig. S5e: simple knock-ins (ChlM-3, -9, -10, -11, -17, -19, -22, -24), knock-in plus integration at another location (ChlM-4), and deletion plus integration at other location (ChlM-21).

## Discussion

We successfully generated a sizable number of CRISPR/Cas9-induced mutations at the *MAA7, CpSRP43* and *ChlM* loci by delivering Cas9 RNP. This is a significant improvement over the first report of a CRISPR/Cas9-induced mutation in *Chlamydomonas*^29^. This improvement may be attributed to the delivery of the Cas9 RNP, as employed successfully in other organisms^28^,^30^,^31^. Vector-driven expression of Cas9 may be toxic to *Chlamydomonas*, probably due to the continuous expression^29^. Delivery of purified Cas9 RNP may have other advantages including technical simplicity and fewer occurrences of off-target events^30^,^42^. From the auxotrophic selection of *MAA7* mutations, we obtained eight small indel mutations that were specifically induced by the *MAA7*-2 sgRNA and the Cas9 protein. Occurrence of these targeted mutations was highest when cells were delivered with the highest amount of Cas9 and *MAA7*-2 sgRNA (Table 1), even though we have not determined optimum conditions that might have to be determined empirically. However, none of the CRISPR/Cas9-induced mutations were frame-shifts; we observed only small indels of three base pairs or multiples. This odd mutation pattern defies a clear explanation, but there are reports of non-random occurrence of mutations induced by CRISPR/Cas9, including the insertion of an A or in-frame deletions^43^,^44^,^45^. The error-prone double strand break (DSB) repair of DNA must be involved in generation of small indel mutations^46^, and the mechanism of DSB repair in *Chlamydomonas* might have played a role in the unique pattern of mutations obtained in our mutagenic screen. We also found that the indels generated at the *ChlM* locus (not counting the vector insertion) tended to be in-frame except for ChlM-24: ChlM-3 and ChlM-22 showed no change; ChlM-9 and ChlM-19 contained in-frame indels. Alternatively, complete loss-of-function mutations could not survive the selection conditions employed in our auxotrophic screens. Even though tryptophan was supplemented in the media, uptake of tryptophan in *Chlamydomonas* is not known, since no true loss-of-function mutations of TSB have been characterized^17^,^33^.

It is interesting to note that the integration of the vector sequences at the Cas9 cut sites of *CpSRP43* and *ChlM* was mediated by NHEJ, since the co-transformed vectors did not contain any sequence from the target genes or the neighboring loci. Knock-in can be achieved by homology-driven recombination (HDR) wherein the vector sequence contains wings of target-flanking sequences, or by NHEJ that does not require any cloning of the wings^24^,^47^,^48^,^49^,^50^,^51^. It is not clear why we obtained predominantly knock-in events. It is possible that DSBs induced by Cas9 facilitated integration of available free ends of co-transformed vectors at the cut site. Our present finding opens up a range of possible applications of NHEJ-mediated knock-in, including insertion of a gene at a specific location without requiring the cloning of homologous sequences.

Conclusively, delivery of Cas9 RNPs improved mutagenic efficiency and specificity of the CRISPR/Cas9 system at the *MAA7, CpSRP43* and *ChlM* loci in *Chlamydomonas*. The efficiency of obtaining targeted mutations was up to 100 fold improvement compared to the first report of CRISPR/Cas9-induced mutagenesis in *Chlamydomonas*^29^. The direct delivery of Cas9 RNPs may have other advantages including minimized off-targeting events, less toxicity of Cas9, and no laborious cloning work compared to the transgenic techniques^30^. In our mutagenic screen targeting the *CpSRP43* locus, sequencing the whole genomes of *cpsrp43* mutants revealed no incidences of off-targeting by using Cas-OFFinder^41^. We also found predominant NHEJ-mediated knock-in events when co-transformed with unrelated vectors used for the selection purpose. New concepts of the targeted transgene integration for stable expression, such as the “safe harbor” technique and other gene manipulations, are already underway in some organisms^47^,^52^. This work can be applied not only to biological research in *Chlamydomonas*, but also to industrial applications of genome editing in other microalgae for large scale production of biofuels and other biomaterials.

## Methods

### Target genes and sgRNA design

We chose target genes based on our ability to identify mutations via auxotrophic or visual means. The *MAA7* gene (GenBank accession: XM_001703345) in *Chlamydomonas* encodes the beta subunit of tryptophan synthase (TSB; XP_001703397), and its mutations can be positively selected using 5-FI^32^,^33^. Mutations in the other two tested genes could be selected based on the lighter colony color arising from reduced chlorophyll synthesis^34^,^35^. The *CpSRP43* gene (GenBank accession: XM_001703652) and *ChlM* gene (GenBank accession: XM_001702328) encodes chloroplast SRP43 (XP_001703704) and Mg-protoporphyrin IX S-adenosyl methionine O-methyl transferase (XP_001702380), respectively. For the latter two genes, we co-transformed cells with a hygromycin-resistance vector and used antibiotic selection to improve the delivery efficiency in *Chlamydomonas*.

The sgRNAs were designed by ToolGen, Inc (Republic of Korea) based on the following inclusion criteria: (1) the target site is located within 50% of coding sequence (CDS) from the N terminus; (2) the out-of-frame score is >60; and (3) the mismatch is 2 or greater. The generated sgRNAs included the following guide RNA sequences. For *MAA7*, *MAA7*-RG1 (5′-UCGAGCGCUCGCGACGGGA-3′) and *MAA7*-RG2 (5′-CAUAGCGACCAUUUGCGUCC-3′); for *CpSPR43*, CpSRP43-RG1 (5′-CGAUUCCGGCCUGCACCGGC-3′); and for *ChlM*, ChlM-RG1 (5′-CCCGCCCGGCUGUGGCCCGG-3′). Their locations along each locus are shown in Fig. 1a–c. All sgRNAs included the common sequence containing the crRNA, tetraloop (underlined) and tracrRNA: GUUUUAGAGCUAGAAAUAGCAAGUUAAAAUAAGGCUAGUCCGUUAUCAACUUGAAAAAGUGGCACCGAGUCGGUGCUUUUUUU^24^. The Cas9 protein contained a nuclear localization signal at the C-terminus, of which sequence is PKKKRKV.

### Microalgal strains and culture conditions

*Chlamydomonas reinhardtii* CC-124 was used as the wild type, and was obtained from the Chlamydomonas Resource Center in the University of Minnesota (http://www.chlamycollection.org/). An RNAi knockdown mutant of *MAA7* (RNAi #33) was kindly provided by Heriberto Cerutti of Nebraska University-Lincoln^17^. Mutant strains of *CpSRP43* CC-4561 (*tla3*-Δ*cpsrp43* cw+ mt+) and CC-4562 (*tla3*-Δ*cpsrp43* cw+ mt−) were obtained from the Chlamydomonas Resource Center, and were used as positive controls for our CRISPR/Cas9-induced mutants^34^. All strains were maintained on Tris-acetate-phosphate (TAP) agar plates [2.42 g/L Tris, 0.375 g/L NH_4_Cl, 0.1 g/L MgSO_4_  7H_2_O, 0.05 g/L CaCl_2_ 2H_2_O, 0.0108 g/L K_2_HPO_4_, 0.0054 g/L KH_2_PO_4_, 1 mL/L glacial acetic acid, and 1 mL/L Hutner’s trace elements (50 g/L Na_2_EDTA 2H_2_O, 22 g/L ZnSO_4_ 7H_2_O, 11.4 g/L H_3_BO_3_, 5.06 g/L MnCl_2_ 4H_2_O, 1.61 g/L CoCl_2_ 6H_2_O, 1.57 g/L CuSO4 5H_2_O, 1.10 g/L (NH_4_)_6_Mo_7_O_24_ 7H_2_O, and 4.99 g/L FeSO_4_ 7H_2_O) at 25 °C under continuous illumination of 50 μmol m^−2^ s^−1^.

### *In vitro* digestion of target DNAs with the Cas9 protein

The activities of the sgRNAs and the Cas9 protein were verified *in vitro* prior to their use *in vivo*. The DNA targets for this assay were produced by PCR using the Instagene Matrix (Bio-Rad, USA). Briefly, cells were harvested by centrifugation and genomic DNAs were extracted according to the instructions provided with the Instagene Matrix. Substrate DNAs encompassing the Cas9 cut site were amplified using 2Χ EF-Taq PCR Pre-mix (Solgent, Republic of Korea). The utilized primers were as follows: for *MAA7*, M2_F and M2_R; for *CpSRP43*, SRP43_2F and SRP43_2R; and for *ChlM*, CrChlM_F1 and CrChlM_R3 (Table S1). The PCR conditions consisted of 35 cycles of 95 °C for 20 sec, 52–55 °C for 40–60 sec and 72 °C for 1 min. The elongation time was extended to 3 min for the longer amplicon of the *CpSRP43* knock-in mutants. The PCR products were purified with a QIAquick Gel Extraction Kit (Qiagen, the Netherlands). For *in vitro* assays, 500 ng of sgRNA and 600 ng of Cas9 protein were pre-mixed at 37 °C for 5 min. For experiments, 100 ng of PCR amplicons and 3.1 NEB buffer (NEB, USA) were added, and the mixtures were incubated at 37 °C for 2 hours. RNaseA (Bioneer, Republic of Korea) was added, and the mixture was incubated at 37 °C for 20 min. 6× STOP solution (0.5 M EDTA, 80% glycerol, and 10% SDS) was added, and the mixture was incubated 37 °C for 20 min. The final products were separated by 0.8% agarose gel electrophoresis.

### Transformation and selection of *C. reinhardtii*

For transformation of *C. reinhardtii*, we performed electroporation using a modification of a previously reported protocol^53^. Cells were cultured under continuous illumination (200 μmol m^−2 ^s^−1^) for 2–3 days to a cell density of 3 × 10^6^ cells/ml. The cells were then concentrated to 3 × 10^8 ^cells/mL in MAX Efficiency Transformation Reagent for Algae (Thermo Fisher Scientific, USA), and electroporation was conducted using an ECM 830 Square Wave Electroporation System (Bio-Rad, USA) and cuvettes with a 0.2-cm gap (BTX, USA). Prior to electroporation, various amounts of Cas9 and sgRNAs were incubated together at 37 °C for 30 min (Table 1), and cells (300 μL) were placed in the cuvette and cooled on ice for 5 min. To enable visual selection of *CpSRP43* and *ChlM* knockout mutants, 1 μg of pCr202 (for *CpSRP43*) or pCr112 (for *ChlM*) were linearized with *Xba*I and added to the mixture, thereby conferring resistance to hygromycin^38^. The electroporation voltage and pulse interval were 250 V and 15 ms, respectively. Electroporated cells were recovered for 16 hours in TAP medium containing 40 mM of sucrose, with agitation (120 rpm) under continuous low light. Mutant cells were selected on TAP agar plates containing 1.5 mM of L-tryptophan and 25 μM of 5-FI (Sigma-Aldrich, USA) for tryptophan auxotrophic selection, or on TAP agar plates with 15 mg/L of hygromycin B (Thermo Fisher Scientific, USA) for co-transformation.

### Complementation of CRISPR/Cas9-induced *MAA7* mutants

To confirm the functional significance of the isolated *MAA7* mutations, we cloned the *MAA7* CDS from CC-124 and *MAA7* 20-1 and performed complementation assays on *MAA7* 20-1 and the RNAi mutant, *MAA7* RNAi #33^17^. Cells were exponentially grown in TAP with shaking at 120 rpm under continuous light of 120 μmol m^−2 ^s^−1^, and total RNAs were isolated using the RNeasy Plant Mini kit (Qiagen, the Netherlands) and the RNase-Free DNase Set (Qiagen, the Netherlands) according to the manufacturer’s instructions. Any remaining DNAs were removed with a DNA-free DNase kit (Ambion, USA), and cDNAs were produced using Superscript III Reverse Transcriptase and oligo (dT)_20_ primers (both from Invitrogen, USA). Individual CDSs were amplified using the Phusion High-Fidelity DNA Polymerase (NEB, USA) and primers I202TSB_3F and I202TSB_3R, and the obtained fragments were cloned into the pCr202 vector^38^ using a Gibson Assembly kit (NEB, USA) according to the manufacturer’s instructions. CDS expression was controlled by the HSP70A and RBCS2 promoter and terminated by the psaD terminator^54^. We added a translation initiation context (AACA) in front of the start codon^55^ and a FLAG-tag sequence (GACTACAAGGACGACGACGACAAG) next to the CDS^56^. Cloned plasmids were delivered to *MAA7* 20-1 and *MAA7* RNAi #33 by electroporation, as described in the previous section. After recovery, transformed cells were spread on TAP agar plates containing 1.5 mM L-tryptophan and 15 mg/L hygromycin B. After 1 week, hygromycin-resistant colonies were subjected to PCR verification of the following: transformant status, as assessed by the presence of the *aphVII* gene (using primers aph72_L and aph72_R); the presence of the transgenic *MAA7* gene, as assessed using TSB flag_4F and TSB flag_4R; and proper amplification of the 18S rDNA (using primers SR6 and SR9) as a positive control. Expression of the transgenic MAA7 protein was confirmed by Western blotting using a rabbit anti-FLAG-tag antibody (diluted 1:1000; Cell Signaling Technology, USA). Clones of Western-positive cells were subjected to complementation assays by growth in 24-well plates (SPL, Republic of Korea) under continuous light (120 μmol m^−2 ^s^−1^) with shaking at 150 rpm for 4 days in the presence or absence of 25 μM 5-FI. Cell growth was monitored using the OD_750 nm_, which was assessed using a SpectraMax M2e Microplate reader (Molecular Devices, USA).

### Chlorophyll fluorescence

Chlorophyll contents were measured using methanol extraction according to published protocols^57^. Briefly, 5 mL of cells were centrifuged at 5,035 × g for 10 min and washed twice with distilled water twice. Pellets were resuspended in the same volume of methanol and incubated in the dark at 4 °C for 1 hour. After centrifugation at 5,035 × g for 10 min, the optical density of supernatant was measured at 652 and 665 nm using a UV-1800 UV-VIS Spectrophotometer (Shimadzu, Japan). The concentrations of chlorophyll a and b were calculated as follow:

Chl a [μg/mL] = −8.0962 × OD_652 nm_ + 16.5169 × OD_665 nm_

Chl b [μg/mL] = 27.4405 × OD_652 nm_ − 12.1688 × OD_665 nm_

The obtained chlorophyll contents were normalized to the cell density, which was measured using a Cellometer AutoT4™ (Nexcelom Bioscience, USA).

### Genomic DNA extraction and whole genome sequencing

Genomic DNA was extracted according to published methods^58^,^59^. Briefly, cells were grown to the stationary phase, harvested, washed with 50 mM EDTA, and resuspended in 150 μL distilled water and 300 μL SDS-EB. DNA was extracted with a mixture of phenol and chloroform (1:1), with repeated applications of the mixture (500 μL) made until the interphase became clear. The DNA was washed with 500 μL chloroform and precipitated with two volumes of ethanol. The obtained DNA pellet was washed with 70% ethanol and air-dried, and the DNA was resuspended in 40 μL of TE buffer. Whole-genome sequencing was performed using an Illumina HiSeq 2000 (Illumina, USA) according to the provided protocol. Genomic DNA sequences were read with a paired-end sequencing platform (Seeders, Republic of Korea). The SolexaQA package software (version 1.13) was used to trim the short read sequences for better quality. More specifically, the DynamicTrim module was used to eliminate poor-quality bases (Phred score < 20) and the LengthSort module was used to exclude short read lengths <25 bp. The trimmed reads were assembled using the SOAPdenovo version 2.04 (http://soap.genomics.org.cn//soapdenovo.html) under default options^60^. The assembled contigs were aligned with the *C. reinhardtii* reference genome v5.5 (http://www.phytozome.net) and with the sequence of the vector pCr202 for *CpSRP43* mutagenic experiments. BLASTN searches were performed using cutoffs of 1e-10 and 90% identity.

### Phylogenetic analysis

Phylogenetic analyses of TSB sequences were performed using the CLC Genomics Workbench version 7.0 (CLC bio, USA). Twenty-three TSB amino acid sequences from organisms representing all five kingdoms were obtained from GenBank (Fig. 2C). For the alignment parameters, the gap-open cost and gap-extension cost were set at 10.0 and 1.0, respectively. The full sequence of the TSB protein, including the Cas9 cut site, is presented in Fig. S1. A phylogenetic tree of the aligned sequences was constructed using the neighbor-joining method, and the Jukes-Cantor model was used to measure protein distance.

### Western blot analysis

To confirm our knockout of the CpSRP43 protein, Western blotting was performed. *C. reinhardtii* cells were grown to the exponential phase, and the cell density was normalized to 10^7^ cells. The cells were harvested, washed once with distilled water, and collected by centrifugation at 13,000 rpm for 10 min. The cell pellets were resuspended with 1.5× Laemmli buffer (62.5 mM Tris-HCl pH 7.6, 7% sodium dodecyl sulfate, 25% glycerol, 5% β-mercaptoethanol, and 0.02% bromophenol blue) and incubated at 100 °C for 5 min. The samples were centrifuged at 13,000 rpm for 5 min, and the supernatants were loaded to Mini-PROTEAN^®^ TGX Stain-Free^TM^ Gel of Any KD (Bio-Rad, USA) and run in 1× TGX buffer. The resolved proteins were transferred to PVDF membranes using the Trans-Blot^®^ Turbo^TM^ System (Bio-Rad, USA). The membranes were blocked with 1× PBS containing 5% skim milk and 0.1% Tween 20 with agitation. The CpSRP43 protein was detected using polyclonal antibodies raised against the antigenic sequence, CERRFKIRWSDGYPTSWEPEE^34^. The β-subunit of ATP synthase (ATPβ) was detected as a loading control. Then, the blots were incubated with horseradish peroxidase (HRP)-conjugated anti-rabbit secondary antibodies (Cell Signaling Technology, USA) for 1 hour, and the results were visualized by chemiluminescent detection using the Clarity Western ECL substrate and a ChemiDoc system (Bio-Rad, USA).

### Southern blot analysis

For Southern blot analysis of the CpSRP43 and ChlM mutants, 10 μg of genomic DNA was digested with *Pst* I/*Nco*I and *Nco*I, respectively, separated by 0.8% agarose gel electrophoresis, and then blotted onto Hybond-N^+^ nylon membranes (Amersham Biosciences, Sweden). 0.3-kb PCR fragments corresponding to the *aphVII*, *CpSRP43* and *ChlM* genes were used as probes. The primers used to generate each probe are listed in Table S1. ^32^P-labeled probes were produced using the Rediprime^TM^ II Random Labeling System (Amersham Biosciences, Sweden) and hybridization was performed following the manufacturer’s instructions. The probe-bound membrane was washed and exposed to a phosphor-imaging plate (IP) for 3 days, and the obtained signals were detected using a Bio-Imaging Analyzer BAS-1800II (Fuji, Japan).

### Semi-quantitative RT-PCR analyses

Total RNA was extracted from WT and ChlM mutants using the TRIzol reagent (Invitrogen, USA) according to the manufacturer’s instructions. Reverse transcription was carried out using 2 μg total RNA, 50 μM oligo (dT)_18_, 200 U M-MLV reverse transcriptase (Promega, USA), 500 μM of each dNTP, and 20 U ribonuclease inhibitor (Thermo Fisher Scientific, USA). For semi-quantitative RT-PCR, cDNAs were amplified with 1.5 U Ex Taq DNA polymerase (TaKaRa, Japan), 100 μM of each dNTP and 10 pmol of each gene-specific primer, using a T1 thermal cycler (Biometra GmbH, Germany). Amplification was performed with 25–40 cycles of 94 °C for 30 s, 56 °C for 30 s, and 72 °C for 30 s. The primers used to amplify the ChlM and IDA5 cDNAs are listed in Table S1.

## Additional Information

**How to cite this article**: Shin, S.-E. *et al*. CRISPR/Cas9-induced knockout and knock-in mutations in *Chlamydomonas reinhardtii*. *Sci. Rep.* **6**, 27810; doi: 10.1038/srep27810 (2016).

## Supplementary Material

Supplementary Information

## Figures and Tables

**Table 1:** Selection and targeting efficiencies in the CRISPR/Cas9 mutagenic screens.

Gene	Type		Amounts of sgRNA (Cas9 protein)				
			Control	10 μg (7.5 μg)	20 μg (15 μg)	40 μg (30 μg)	Total
*MAA7*-1	Selection	Colonies	0	1	2	–a	3
		Efficiency (cell^−1^)^b^	0	1.1 × 10^−8^	6.7 × 10^−8^	–	3.3 × 10^−8^
	Targeting	Colonies	0	0	0	–	0 (0%)^c^
		Efficiency (cell^−1^)	0	0	0	–	0
*MAA7*-2	Selection	Colonies	1	6	5	9	20
		Efficiency (cell^−1^)	1.1 × 10^−8^	6.7 × 10^−8^	5.6 × 10^−8^	1.0 × 10^−7^	2.2 × 10^−7^
	Targeting	Colonies	0	0	2	6	8 (40%)
		Efficiency (cell^−1^)	0	0	2.2 × 10^−8^	6.7 × 10^−8^	8.9 × 10^−8^
*CpSRP43*	Selection	Colonies	65	34	108	76	218
		Efficiency (cell^−1^)	7.2 × 10^−7^	3.8 × 10^−7^	1.2 × 10^−6^	8.4 × 10^−7^	2.4 × 10^−6^
	Targeting	Colonies	0	3	0	0	3 (1.4%)
		Efficiency (cell^−1^)	0	3.3 × 10^−8^	0	0	3.3 × 10^−8^
*ChlM*	Selection	Colonies	–	6000	–	–	6000
		Efficiency (cell^−1^)	–	3.0 × 10^−5^	–	–	3.0 × 10^−5^
	Targeting	Colonies	–	10	–	–	10 (0.17%)
		Efficiency (cell^−1^)	–	5.0 × 10^−8^	–	–	5.0 × 10^−8^

^a^Not tested.

^b^The efficiency was calculated by dividing the number of colonies with that of cells used for electroporation.

^c^Targeting percentage was calculated by dividing the number of targeted colonies with that of selected colonies.
