# Supplementary material for: CRISPR/Cas9-induced knockout and knock-in mutations in Chlamydomonas reinhardtii
Source: Sci Rep. 2016 Jun 13;6:27810. doi: 10.1038/srep27810 (PMC4904240; doi:10.1038/srep27810)
Supplement: Supplementary Information [file srep27810-s1.pdf]

Supplementary Information for Scientific Reports

CRISPR/Cas9-induced knockout and knock-in mutations in *Chlamydomonas reinhardtii*

Sung-Eun Shin[a,§], Jong-Min Lim[b,§], Hyun Gi Koh[a], Eun Kyung Kim[h], Nam Kyu Kang[a], Seungjib Jeon[a], Sohee Kwon[a], Won-Sub Shin[a], Bongsoo Lee[a], Kwon Hwangbo[b,c], Jungeun Kim[d,e], Sung Hyeok Ye[d,f], Jae-Young Yun[d], Hogyun Seo[g], Hee-Mock Oh[b], Kyung-Jin Kim[g], Jin-Soo Kim[d,e], Won-Joong Jeong[b*], Yong Keun Chang[a,h*], Byeong-ryool Jeong[a*]

[a] Department of Chemical and Biomolecular Engineering, Korea Advanced Institute of Science and Technology (KAIST), Daejeon 34141, Republic of Korea

[b] Korea Research Institute of Bioscience and Biotechnology (KRIBB), Daejeon 34141, Republic of Korea

[c] Department of Biological Science, Chungnam National University (CNU), Daejeon 34134, Republic of Korea

[d] Center for Genome Engineering, Institute for Basic Science (IBS), Seoul 08826, Republic of Korea

[e] Department of Chemistry, Seoul National University (SNU), Seoul 08826, Republic of Korea

[f] Basic science, IBS school, Korea University of Science and Technology (UST), Seoul 08826, Republic of Korea

[g] School of Life Sciences, KNU Creative BioResearch Group, Kyungpook National University (KNU), Daegu 41566, Republic of Korea

[h] Advanced Biomass R&D Center (ABC), KAIST, Daejeon 34141, Republic of Korea

§: Both authors contributed equally to this work.

[*]Corresponding authors:

Byeong-ryool Jeong is to be contacted at Tel.: +82-42-350-3964, Fax: +82-42-350-8858, E-mail: bjeong@kaist.ac.kr; Yong Keun Chang: +82-42-350-3924, changyk@kaist.ac.kr; Won-Joong Jeong: +82-42-860-4462, wonjoong@kribb.re.kr.

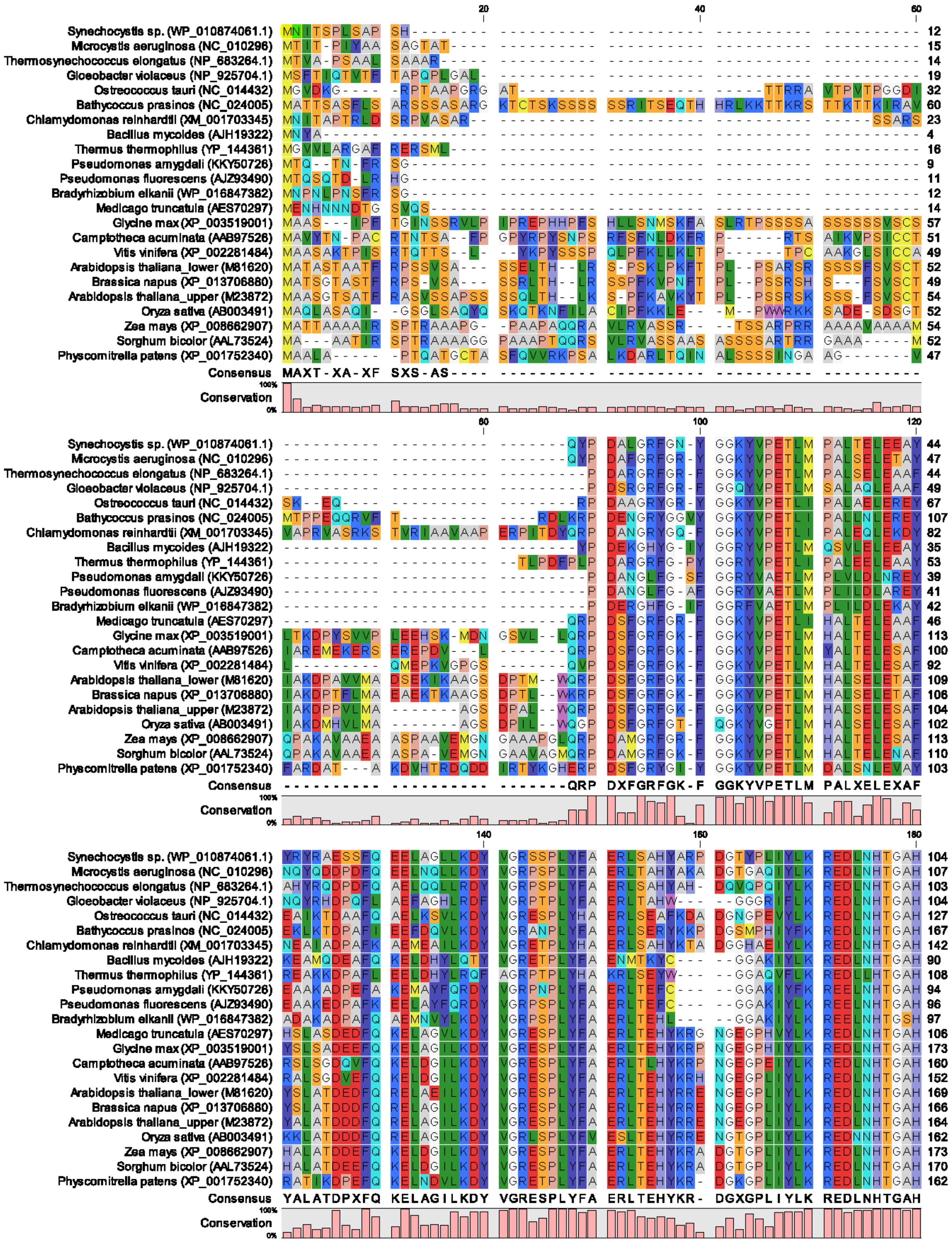
Synechocystis sp. (WP_010874061.1)
Microcystis aeruginosa (NC_010296)
Thermosynechococcus elongatus (NP_683264.1)
Gloeobacter violaceus (NP_925704.1)
Ostreococcus tauri (NC_014432)
Bathycoccus prasinos (NC_024005)
Chlamydomonas reinhardtii (XM_001703345)
Bacillus mycoides (AJH19322)
Thermus thermophilus (YP_144361)
Pseudomonas amygdali (KKY50726)
Pseudomonas fluorescens (AJZ93490)
Bradyrhizobium elkanii (WP_016847382)
Medicago truncatula (AES70297)
Glycine max (XP_003519001)
Camptotheca acuminata (AAB97526)
Vitis vinifera (XP_002281484)
Arabidopsis thaliana_lower (M81620)
Brassica napus (XP_013706880)
Arabidopsis thaliana_upper (M23872)
Oryza sativa (AB003491)
Zea mays (XP_008662907)
Sorghum bicolor (AAL73524)
Physcomitrella patens (XP_001752340)
Consensus
MAXT-XA-XF SXS-AS----
Consensus
-------QRP DXFGRFGK-F GGKYVPETLM PALXELEXAF
Consensus
YALATDPXFQ KELAGILKDY VGRESPLYFA ERLTEHYKR- DGXGPLIYLK REDLNHTGAH
Conservation
100%
0%
20
40
60
80
100
120
140
160
180

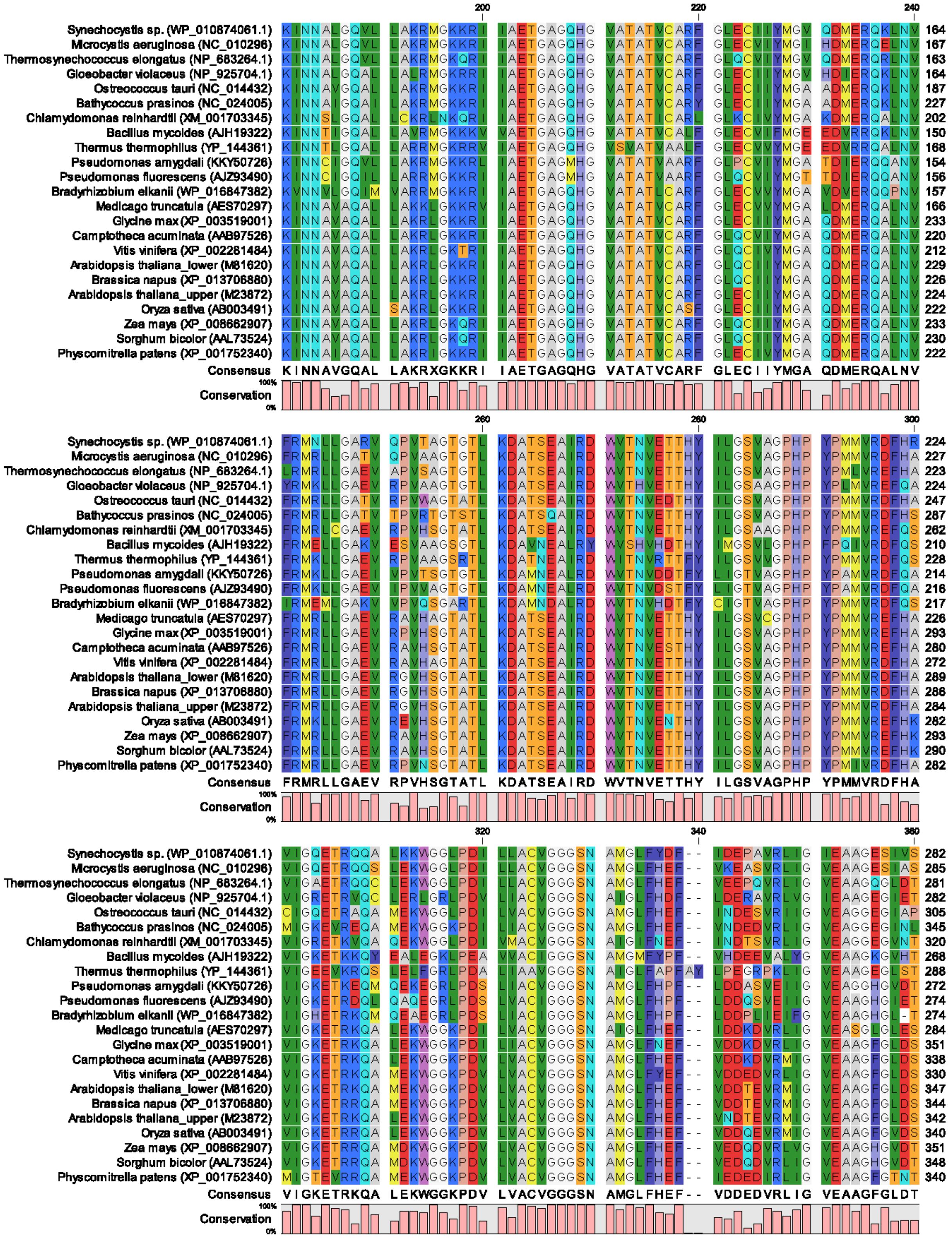
Synechocystis sp. (WP_010874061.1)
Microcystis aeruginosa (NC_010296)
Thermosynechococcus elongatus (NP_683264.1)
Gloeobacter violaceus (NP_925704.1)
Ostreococcus tauri (NC_014432)
Bathycoccus prasinos (NC_024005)
Chlamydomonas reinhardtii (XM_001703345)
Bacillus mycoides (AJH19322)
Thermus thermophilus (YP_144361)
Pseudomonas amygdali (KKY50726)
Pseudomonas fluorescens (AJZ93490)
Bradyrhizobium elkanii (WP_016847382)
Medicago truncatula (AES70297)
Glycine max (XP_003519001)
Camptotheca acuminata (AAB97526)
Vitis vinifera (XP_002281484)
Arabidopsis thaliana_lower (M81620)
Brassica napus (XP_013706880)
Arabidopsis thaliana_upper (M23872)
Oryza sativa (AB003491)
Zea mays (XP_008662907)
Sorghum bicolor (AAL73524)
Physcomitrella patens (XP_001752340)
Consensus
Conservation
100%
0%
200
220
240
KINNAVGQAL LAKRXGKKRI IAETGAGQHG VATATVCARF GLECIIYMGA QDMERQALNV
260
280
300
FRMRLLGAEV RPVHSGTATL KDATSEAIRD WVTNVETTHY ILGSVAGPHP YPMMVRDFHA
320
340
360
VIGKETRKQA LEKWGGKPDV LVACVGGGSN AMGLFHEF-- VDDEDVRLIG VEAAGFGLDT

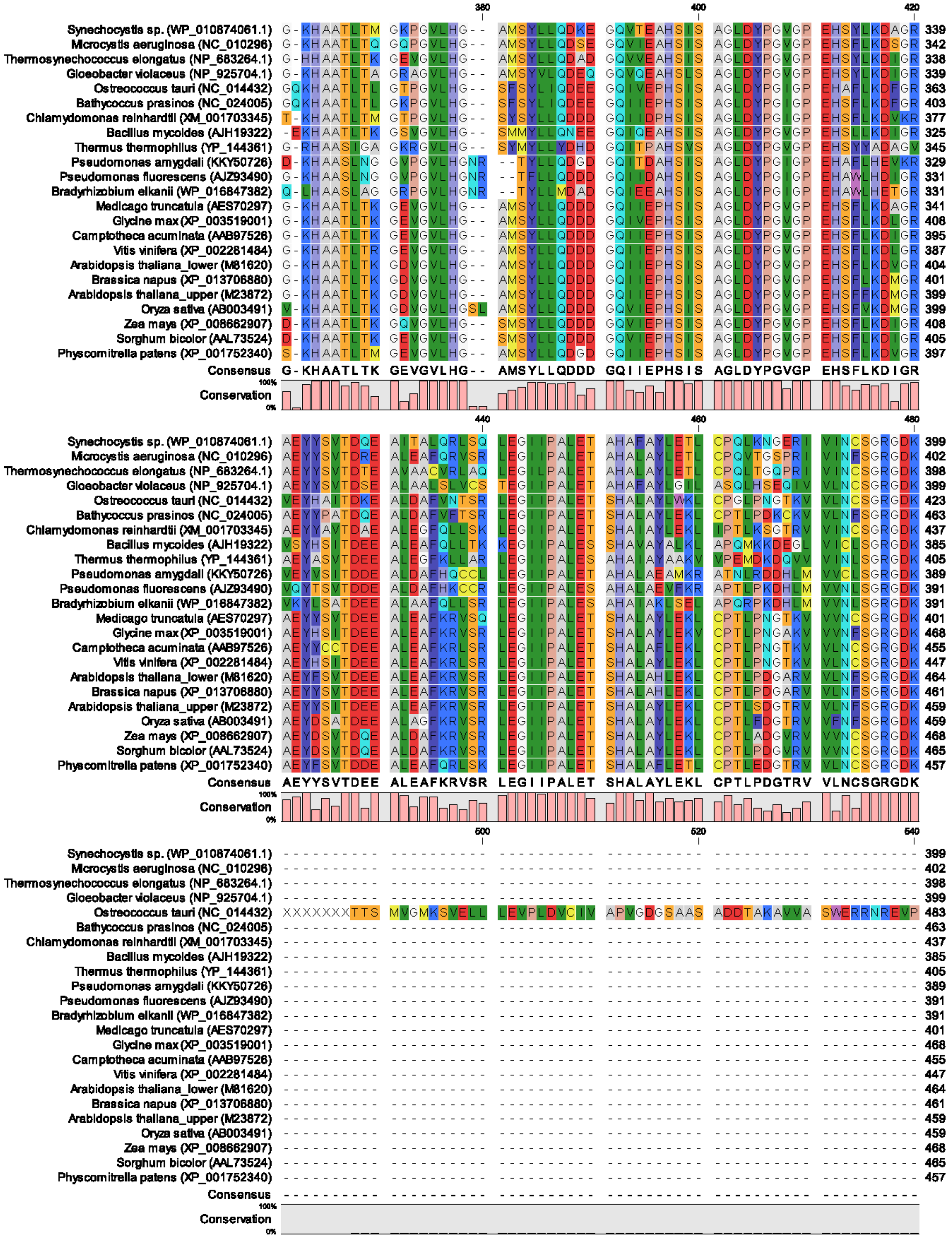

380
400
420
Synechocystis sp. (WP_010874061.1)
Microcystis aeruginosa (NC_010296)
Thermosynechococcus elongatus (NP_683264.1)
Gloeobacter violaceus (NP_925704.1)
Ostreococcus tauri (NC_014432)
Bathycoccus prasinos (NC_024005)
Chlamydomonas reinhardtii (XM_001703345)
Bacillus mycoides (AJH19322)
Thermus thermophilus (YP_144361)
Pseudomonas amygdali (KKY50726)
Pseudomonas fluorescens (AJZ93490)
Bradyrhizobium elkanii (WP_016847382)
Medicago truncatula (AES70297)
Glycine max (XP_003519001)
Camptotheca acuminata (AAB97526)
Vitis vinifera (XP_002281484)
Arabidopsis thaliana_lower (M81620)
Brassica napus (XP_013706880)
Arabidopsis thaliana_upper (M23872)
Oryza sativa (AB003491)
Zea mays (XP_008662907)
Sorghum bicolor (AAL73524)
Physcomitrella patens (XP_001752340)
Consensus G-KHAATLTK GEVGVLHG-- AMSYLLQDDD GQIIEPHSIS AGLDYPGVGP EHSFLKDIGR
Conservation
440
460
480
Consensus AEYYSVTDEE ALEAFKRVSR LEGIIPALET SHALAYLEKL CPTLPDGTRV VLNCSGRGDK
Conservation
500
520
540
Consensus
Conservation

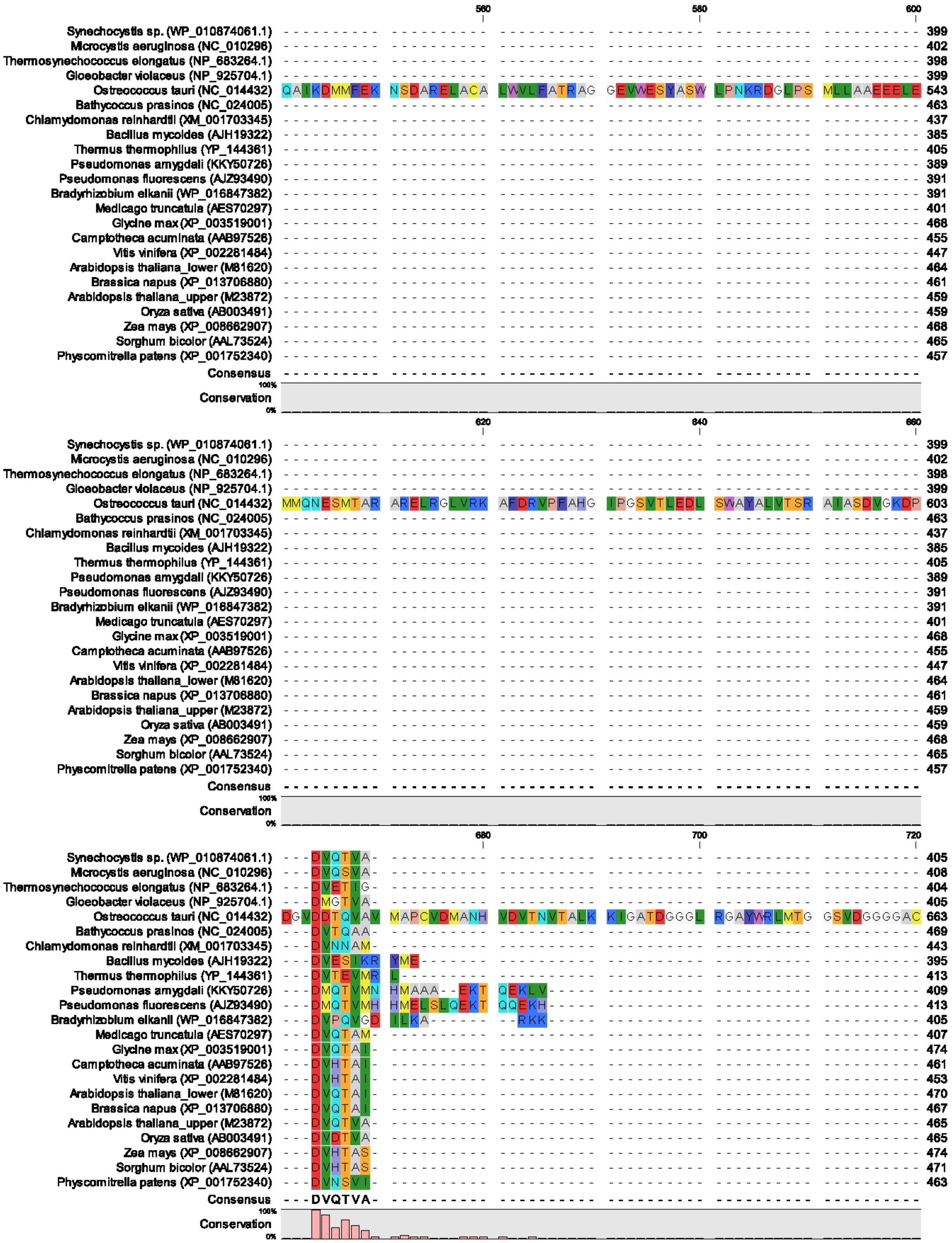
560
580
600
Synechocystis sp. (WP_010874061.1) 399
Microcystis aeruginosa (NC_010296) 402
Thermosynechococcus elongatus (NP_683264.1) 398
Gloeobacter violaceus (NP_925704.1) 399
Ostreococcus tauri (NC_014432) 543
Bathycoccus prasinos (NC_024005) 463
Chlamydomonas reinhardtii (XM_001703345) 437
Bacillus mycoides (AJH19322) 385
Thermus thermophilus (YP_144361) 405
Pseudomonas amygdali (KKY50726) 389
Pseudomonas fluorescens (AJZ93490) 391
Bradyrhizobium elkanii (WP_016847382) 391
Medicago truncatula (AES70297) 401
Glycine max (XP_003519001) 466
Camptotheca acuminata (AAB97526) 455
Vitis vinifera (XP_002281484) 447
Arabidopsis thaliana_lower (M81620) 464
Brassica napus (XP_013706880) 461
Arabidopsis thaliana_upper (M23872) 459
Oryza sativa (AB003491) 459
Zea mays (XP_008662907) 468
Sorghum bicolor (AAL73524) 465
Physcomitrella patens (XP_001752340) 457
Consensus
Conservation
620
640
660
Ostreococcus tauri (NC_014432) 603
680
700
720
Synechocystis sp. (WP_010874061.1) 405
Microcystis aeruginosa (NC_010296) 408
Thermosynechococcus elongatus (NP_683264.1) 404
Gloeobacter violaceus (NP_925704.1) 405
Ostreococcus tauri (NC_014432) 663
Bathycoccus prasinos (NC_024005) 469
Chlamydomonas reinhardtii (XM_001703345) 443
Bacillus mycoides (AJH19322) 395
Thermus thermophilus (YP_144361) 413
Pseudomonas amygdali (KKY50726) 409
Pseudomonas fluorescens (AJZ93490) 413
Bradyrhizobium elkanii (WP_016847382) 405
Medicago truncatula (AES70297) 407
Glycine max (XP_003519001) 474
Camptotheca acuminata (AAB97526) 461
Vitis vinifera (XP_002281484) 453
Arabidopsis thaliana_lower (M81620) 470
Brassica napus (XP_013706880) 467
Arabidopsis thaliana_upper (M23872) 465
Oryza sativa (AB003491) 465
Zea mays (XP_008662907) 474
Sorghum bicolor (AAL73524) 471
Physcomitrella patens (XP_001752340) 463
Consensus DVQTVA
Conservation

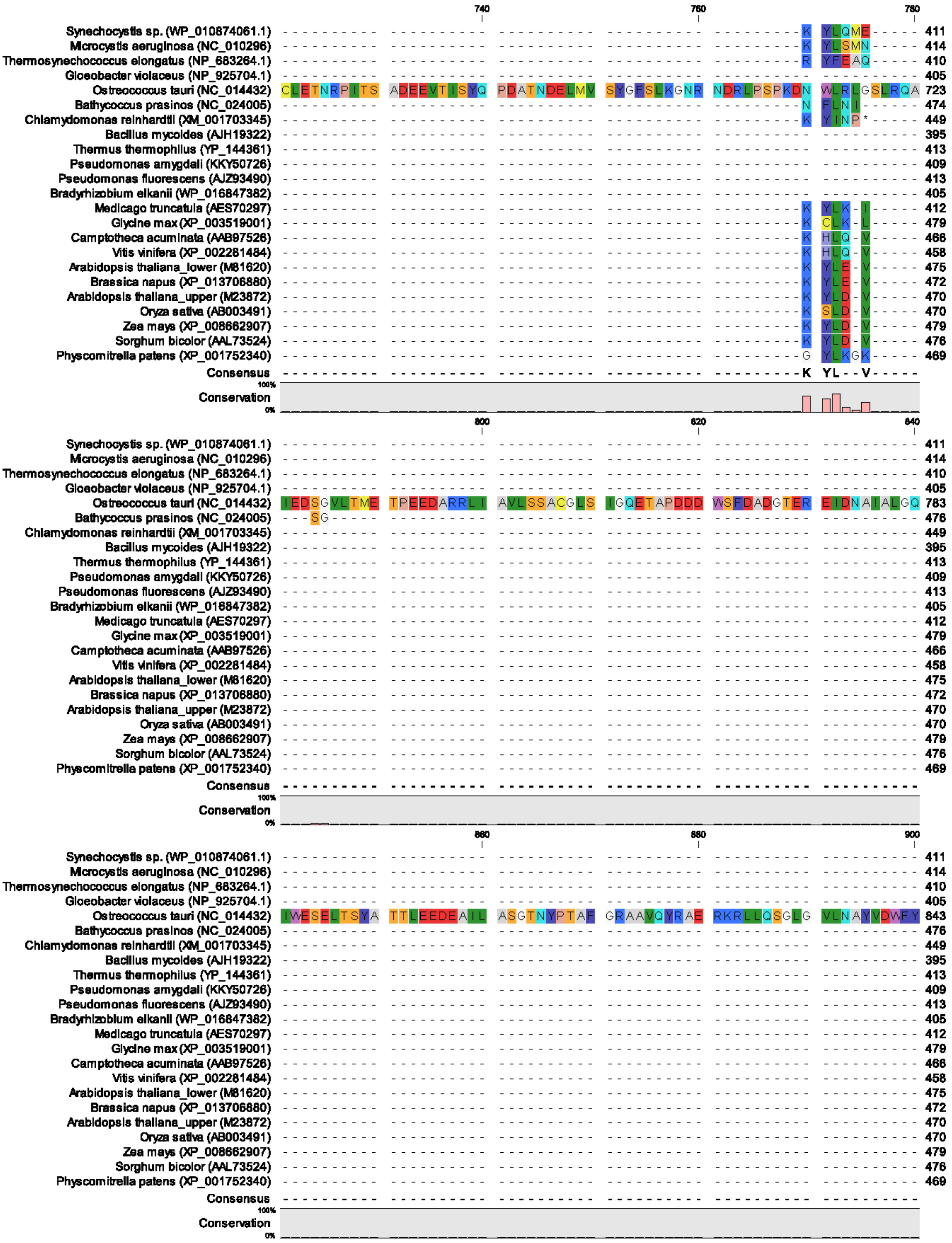
740
760
780
Synechocystis sp. (WP_010874061.1)
Microcystis aeruginosa (NC_010296)
Thermosynechococcus elongatus (NP_683264.1)
Gloeobacter violaceus (NP_925704.1)
Ostreococcus tauri (NC_014432)
Bathycoccus prasinos (NC_024005)
Chlamydomonas reinhardtii (XM_001703345)
Bacillus mycoides (AJH19322)
Thermus thermophilus (YP_144361)
Pseudomonas amygdali (KKY50726)
Pseudomonas fluorescens (AJZ93490)
Bradyrhizobium elkanii (WP_016847382)
Medicago truncatula (AES70297)
Glycine max (XP_003519001)
Camptotheca acuminata (AAB97526)
Vitis vinifera (XP_002281484)
Arabidopsis thaliana_lower (M81620)
Brassica napus (XP_013706880)
Arabidopsis thaliana_upper (M23872)
Oryza sativa (AB003491)
Zea mays (XP_008662907)
Sorghum bicolor (AAL73524)
Physcomitrella patens (XP_001752340)
Consensus
Conservation
800
820
840
860
880
900

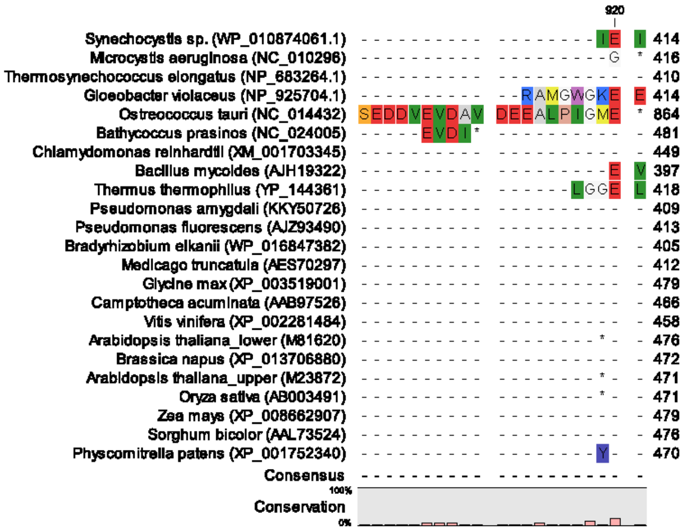

Supplementary Fig. 1. Alignment of the full-length TSB amino acid sequences from organisms representing three kingdoms.

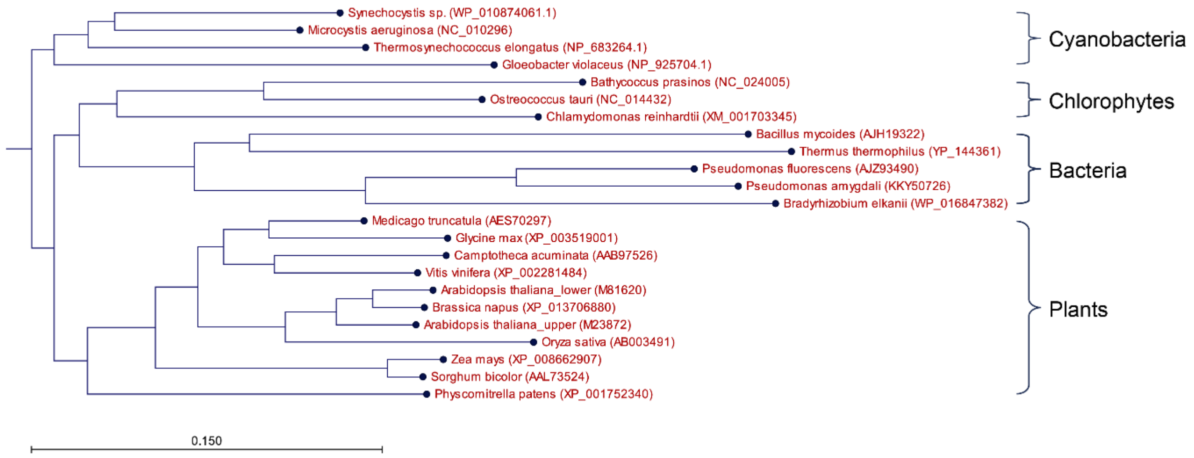

Supplementary Fig. 2. Phylogenetic tree of the TSB amino acid sequences.

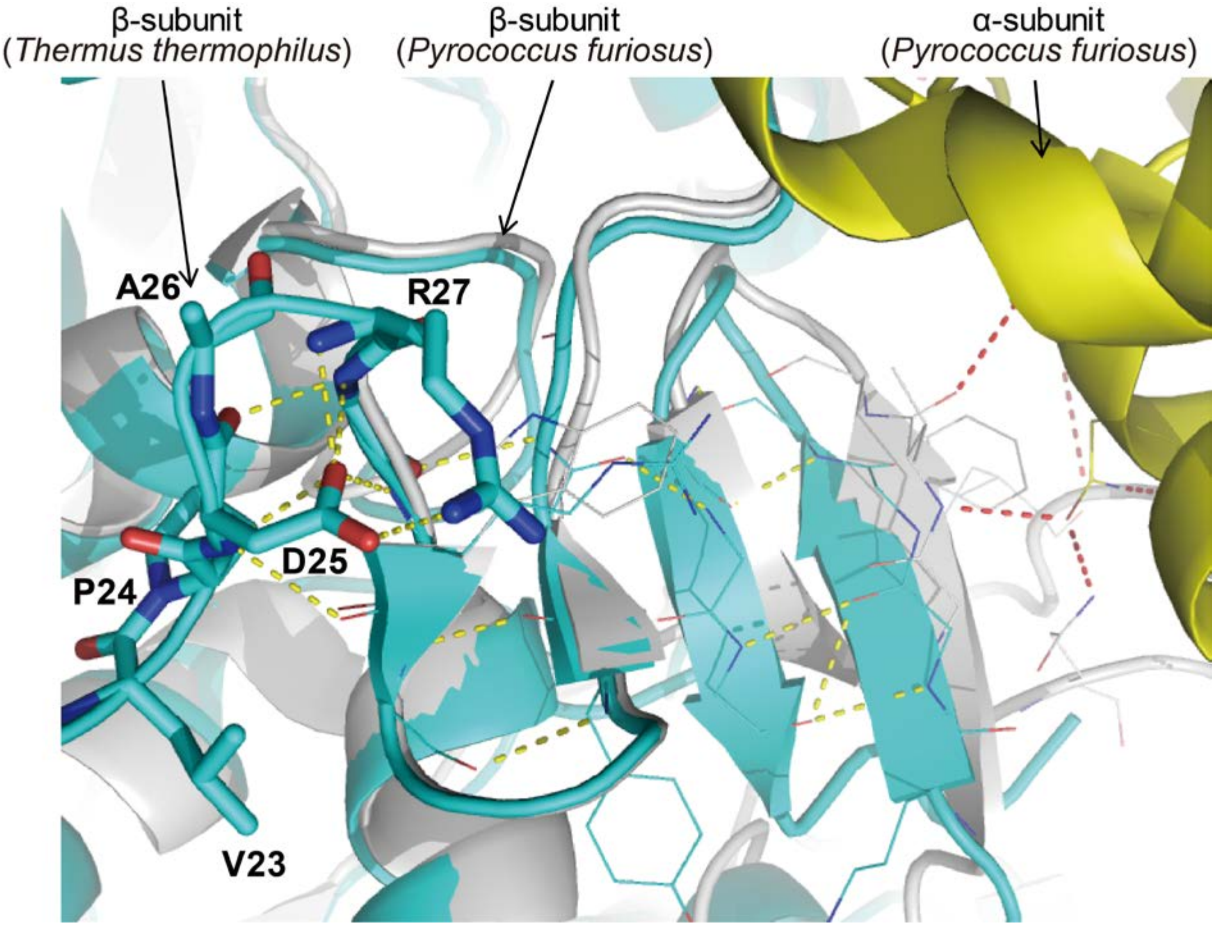

Supplementary Fig. 3. Crystal structures of tryptophan synthases and the region corresponding to the RPDAN motif. The α and the β subunits of the tryptophan synthase from *Pyrococcus furiosus* (PDB CODE 1WDW) is shown as a cartoon diagram with yellow and gray colors, respectively. The β subunit from *Thermus thermophilus* (PDB CODE 1X1Q) is superposed with the corresponding subunit of the tryptophan synthase from *P. furiosus*, and is shown in cyan. The β subunit of the tryptophan synthase from *T. thermophilus* has an LPDAR (stick model) sequence at the position of the RPDAN motif of *C. reinhardtii*. The hydrogen bonds forming the β-sheet are shown as dotted yellow lines, and those forming the interface between the two subunits are shown as dotted red lines.

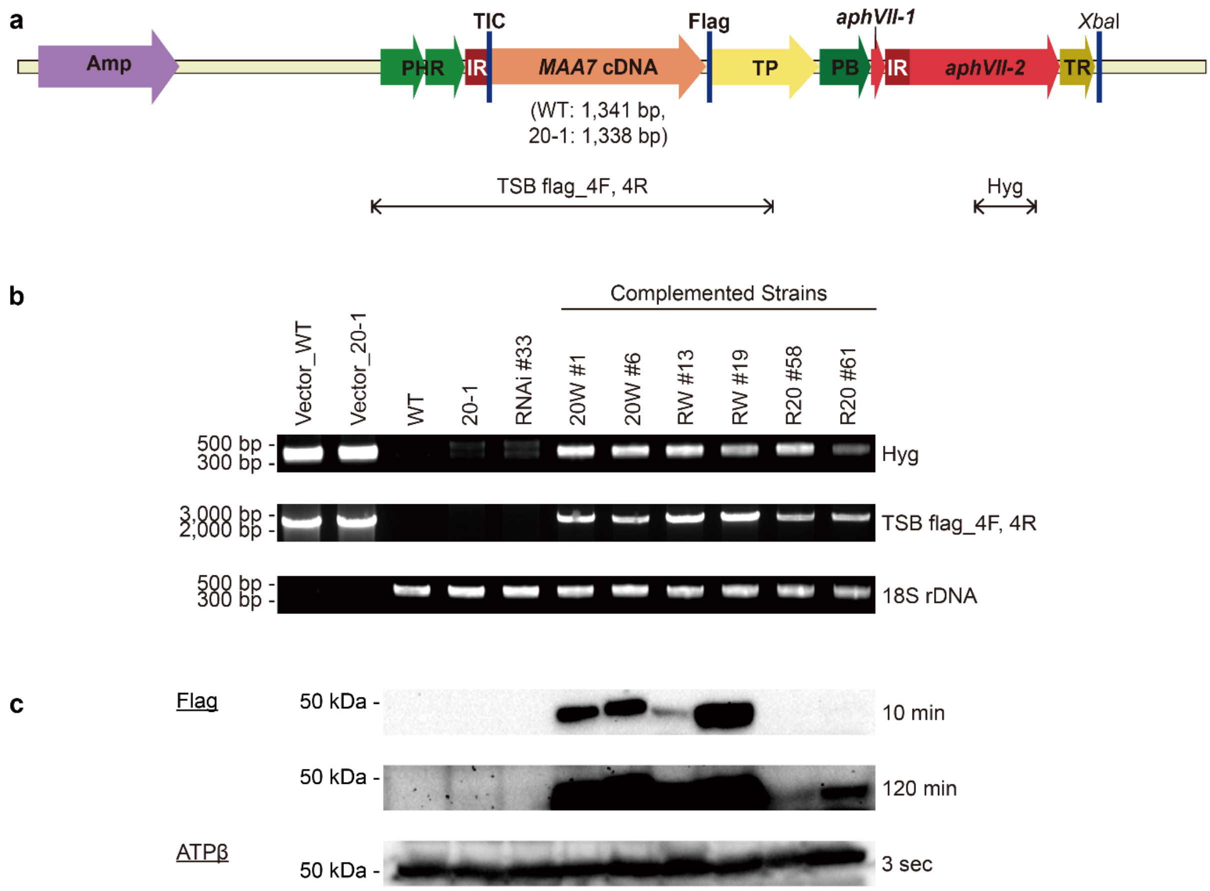

Supplementary Fig. 4. Select complemented strains of MAA7 mutants. (**a**) Schematic diagram of the vector used for complementation. Abbreviations: PHR, HSP70A-RBCS2 promoter; IR, RBCS2 intron; TIC, translation initiation context; TP, PsaD terminator; PB, beta2-tub promoter; and TR, RBCS2 terminator. (**b**) PCR confirmation of the integration of vector sequences into the complemented strains. The PCR amplicons for the hygromycin resistance region and 18S rDNA were 387 bp and 380 bp, respectively, while those encompassing the MAA7 cDNA and Flag tag were 2,477 bp for WT and 2,474 bp for MAA7 20-1. (**c**) Western blotting of FLAG-tagged MAA7 proteins from complemented strains. The expected sizes of FLAG-tagged MAA7 were 49.6 kDa for WT and 49.5 kDa for MAA7 20-1. ATPβ (the beta subunit of ATP synthase; 53.9 kDa) was used as a loading control.

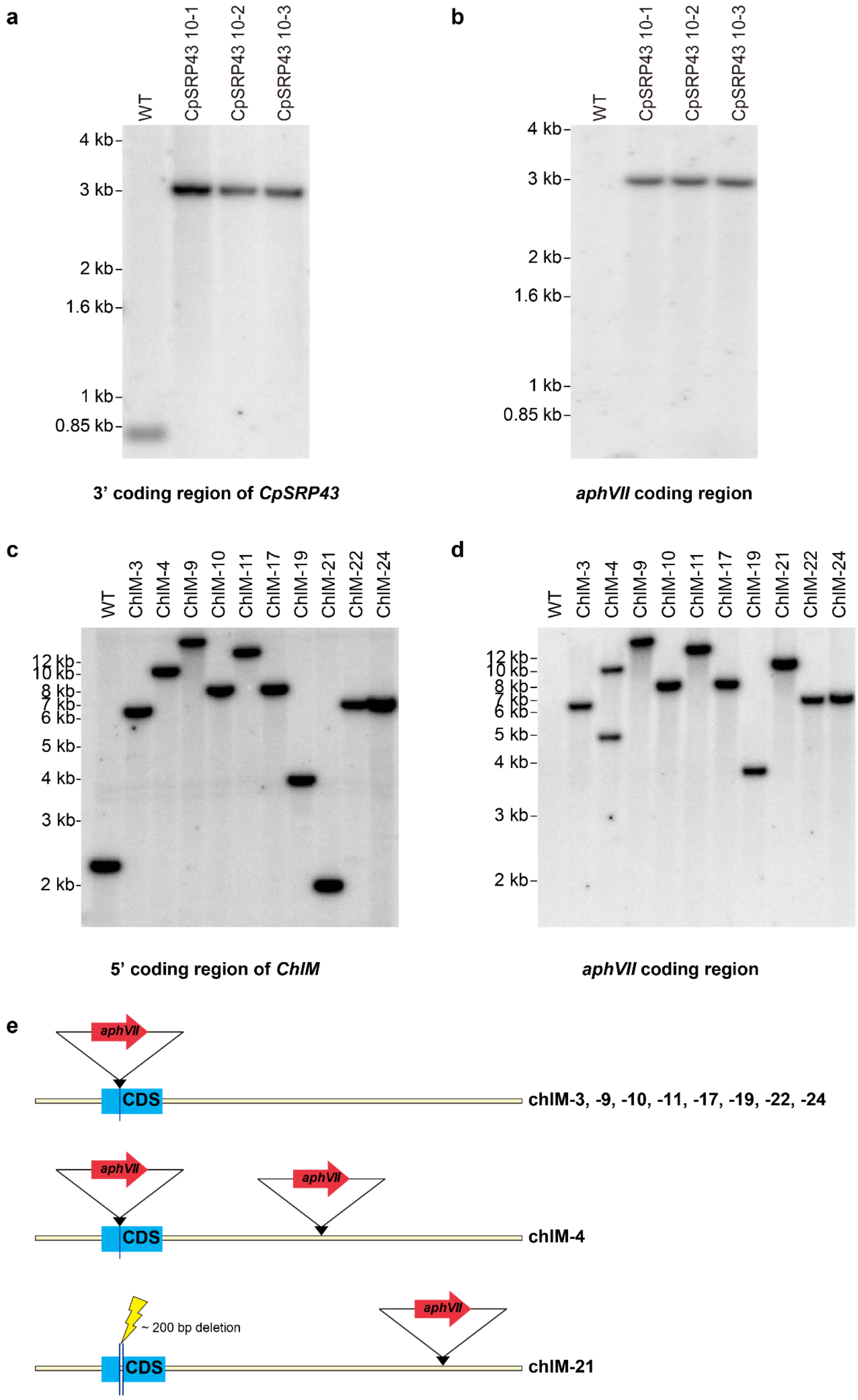
a
WT
CpSRP43 10-1
CpSRP43 10-2
CpSRP43 10-3
4 kb
3 kb
2 kb
1.6 kb
1 kb
0.85 kb
3' coding region of *CpSRP43*
b
WT
CpSRP43 10-1
CpSRP43 10-2
CpSRP43 10-3
4 kb
3 kb
2 kb
1.6 kb
1 kb
0.85 kb
aphVII coding region
c
WT
ChlM-3
ChlM-4
ChlM-9
ChlM-10
ChlM-11
ChlM-17
ChlM-19
ChlM-21
ChlM-22
ChlM-24
12 kb
10 kb
8 kb
7 kb
6 kb
5 kb
4 kb
3 kb
2 kb
5' coding region of *ChlM*
d
WT
ChlM-3
ChlM-4
ChlM-9
ChlM-10
ChlM-11
ChlM-17
ChlM-19
ChlM-21
ChlM-22
ChlM-24
12 kb
10 kb
8 kb
7 kb
6 kb
5 kb
4 kb
3 kb
2 kb
aphVII coding region
e
aphVII
CDS
chlM-3, -9, -10, -11, -17, -19, -22, -24
aphVII
aphVII
CDS
chlM-4
~ 200 bp deletion
aphVII
CDS
chlM-21

Supplementary Fig. 5. Southern blot analyses of the CpSRP43 and ChlM mutants. Genomic DNAs of the *CpSRP43* mutants were digested with *Pst*I and *Nco*I and subjected to Southern blotting with probes against the 3' coding region of *CpSRP43* (**a**) and the *aphVII* coding region (**b**). Genomic DNA of *ChlM* mutants was digested by *Nco*I and subjected to Southern blotting with probes against the 5' region of *ChlM* (**c**) and the *aphVII* coding region (**d**). Main classes of knock-in mutations in the ChlM locus were indicated in schematic drawing (**e**).

Supplementary Table 1. The primers used in our CRISPR/Cas9 systems.

Gene	Primer	Sequence 5'-3'	Purpose	Size (bp)
MAA7	M2_F<br>M2_R	CAACGTTTGCCGTTGGTATG<br>TTCAGGATAGCCTCCATCTCC	Amplifying Cas9 cut site of *MAA7* (*in vitro*)	783
	I202TSB_3F<br>I202TSB_3R	GTCGCCGTTTCCATTTGCAGGATGGCCATGGAAGCTTGATAACAATGAACATCACTGCCCCTACTC<br>TGATACAGGCGGTCCAATCTGAATTGGGCTGCAGGAATTCGATTCACTTGTCGTCGTCGTCCTTGTAGTCAGGGTTGATGTACTTCATGGCGTT	Vector construction for complementation of *MAA7*	1,455
	aph 72_L<br>aph 72_R	CCCCCATTCCGAGGTCTTCC<br>AAGGTGAAGGCGAGCAGTTC	Confirmation of complemented *MAA7* strains (Hygromycin resistant)	387
	TSB flag_4F<br>TSB flag_4R	GCGCAATTAACCCTCACT<br>CAAACTCTCACATGGCCT	Confirmation of complemented *MAA7* strains (transgenic *MAA7* gene)	2,477 for WT<br>2,474 for *MAA7* 20-1
	SR6<br>SR9	GTCAGAGGTGAAATTCTTGG<br>AACTAAGAACGGCATGCAC	Confirmation of complemented *MAA7* strains (18S rDNA)	380
CpSRP43	SRP43_2F<br>SRP43_2R	TATTAGGGCATCGACAGG<br>TGGAAGAATTGGAATGGG	Amplifying Cas9 cut site of *CpSRP43* (in vitro)	795
	SRP43_1F<br>PRR2	CAAATACCATCGGCGTGTGG<br>ACTGACCCACAGCTGTCCTAAGCC	Region A	509
	PRR2_RC<br>hpt1579_R	GGCTTAGGACAGCTGTGGGTCAGT<br>TACCGCTTCAGCACTTGAGA	Region B	1,662
	aph72_L<br>SRP43_1R	CCCCCATTCCGAGGTCTTCC<br>GCGACACCTCGTGAAGCATA	Region C	1,089
	aph7_1471F<br>aph7_1114R	TGGTGCAACTGCATCTCAAC<br>CCAGAGGAACTGCGCCAGTTCCT	Southern blot of *aphVII* probe	203
	SRP43_4F<br>SRP43_4R	AAATGGAAGGTGAGAGCTGG<br>GTCAGATCCGTCCTGTGGAA	Southern blot of *CpSRP43* probe	305
ChlM	CrChlM_F1<br>CrChlM_R3	GTGTTGCAGAGGTGTCTCAGGTTGCCAACA<br>CCGTCTCGCCGTAGATCTTGCGCCA	Amplifying Cas9 cut site of *ChlM* (*in vitro*)	1,010
	aph7_1471F<br>aph7_1114R		Hygromycin resistant	203
	CrChlM_F<br>aph7_1471F	TCGCTGCGTGGCGGAAGCACTA	Region D in ChlM-3, 22, 24	2,000 for ChlM-3<br>1,700 for ChlM-22<br>1,800 for ChlM-24
	CrChlM-F1<br>psaD_ter_R	 GGTCTTCTCAATGGGTGTGGCACGT	Region D in ChlM-9	500
	CrChlM_F1<br>aph7_1471F		Region D in ChlM-10, 17	500
	CrchlM_F3<br>aph7_R2	GCATGGCAAGCTGGCGTCAGCA<br>TTCCGGTCGGTCGTGCCGTCCAT	Region D in ChlM-19	650
	psaD_ter_R1<br>CrChlM_R1	CCTGTGGCTAATTGACCGTG<br>GCACTCCTGCACCCTTCTCACCGCT	Region E in ChlM-3, 22, 24	400 for ChlM-3<br>1,000 for ChlM-22, 24
	aph7_1471F<br>CrChlM_R1		Region E in ChlM-9, 19	700 for ChlM-9<br>500 for ChlM-19
	CrChlM_F1<br>CrChlM_R2	 CCGAGGTAAAAGGCTTGGTGTTTAGGCA	Southern blot of *ChlM* probe	257
	ChlM_RT_F<br>ChlM_RT_R	ATGCAGTGCCTAAACACCAAGCCT<br>CCGTCTCGCCGTAGATCTTGCGCCA	RT-PCR (*ChlM*)	361
	IDA5_F<br>IDA5_R	ATCGTGCGCGACATCAAGGA<br>TACTCGGACTTGGCGATCCA	RT-PCR (loading control)	464
